# Hydrodynamic Limit of Condensing Two-Species Zero Range Processes with Sub-critical Initial Profiles

**DOI:** 10.1007/s10955-017-1827-6

**Published:** 2017-07-01

**Authors:** Nicolas Dirr, Marios G. Stamatakis, Johannes Zimmer

**Affiliations:** 1 School of Mathematics, Cardiff University, Cardiff, CF24 4AG UK; 2 Department of Mathematical Sciences, University of Bath, Bath, BA2 7AY UK

**Keywords:** Hydrodynamic limit, Zero range processes, Condensing zero range processes, Comparison principles for systems of PDEs

## Abstract

Two-species condensing zero range processes (ZRPs) are interacting particle systems with two species of particles and zero range interaction exhibiting phase separation outside a domain of sub-critical densities. We prove the hydrodynamic limit of nearest neighbour mean zero two-species condensing ZRP with bounded local jump rate for sub-critical initial profiles, i.e., for initial profiles whose image is contained in the region of sub-critical densities. The proof is based on H.T. Yau’s relative entropy method, which relies on the existence of sufficiently regular solutions to the hydrodynamic equation. In the particular case of the species-blind ZRP, we prove that the solutions of the hydrodynamic equation exist globally in time and thus the hydrodynamic limit is valid for all times.

## Introduction

In this article, we derive the hydrodynamic limit of a system of two interacting particle systems, specifically two-species zero range processes (ZRPs). The motivation for this study is that hydrodynamic limits provide effective descriptions of large scale interacting particle systems. There is a now good understanding of this limit passage for a range of particle processes leading to one hydrodynamic limit equation. In particular, Kipnis and Landim [19] establish the hydrodynamic behaviour for the one-species zero range process, using the entropy method of Guo, Papanicolaou and Varadhan [17]. For systems, however, this limit passage is less well studied, and several tools available for single equations are no longer available, as explained in more detail below. In particular, many systems where a hydrodynamic passage would be of interest both in its own right and as a tool to understand the limiting system of partial differential equations (PDEs) are currently inaccessible to the methods available; the full Patlak Keller-Segel system [18] modelling the evolution of cells or bacteria guided by the concentration of a chemical substance is an example. Yet, there are several recent studies focusing on different models of interacting particle systems. One avenue is to derive equations which incorporate aspects of underlying models, be it by considering the motion of cells only in a stationary, but random, environment mimicking the chemical [14], or by an equation with a singular potential related to a Green’s function describing the solution of a second equation [11]. The hydrodynamic limit system of an active exclusion process modelling active matter has been recently derived using a two-block estimate and non-gradient estimates [3].

Another approach is to study systems related to underlying zero-range processes (ZRPs) of several species to obtain a limiting system, and this is the approach we pursue here. The focus on ZRPs can be motivated by their nature as a toy model of an interacting particle system. We consider a system of two zero-range processes but the extension to *n* types is straightforward. Each ZRP is a process on a lattice where particles jump from one site to another according to a jump rate function depending on the number of the two species of particles on this site only (hence the name zero range).

The hydrodynamic limit in the Eulerian scaling $t\mapsto tN$ of asymmetric many-species ZRPs with product and translation invariant equilibrium states has been studied in [16]. The hydrodynamic limit in the parabolic scaling $t\mapsto tN^2$ for a class of processes not satisfying the assumptions of [16] has also recently been studied [26]; there one type of particles performs a random walk and influences the other type, which is a process of ZRP type. In general, establishing hydrodynamic limits for systems of equations rigorously is a hard problem, with few known results so far. To name a few, the hydrodynamic limit of a two-species simple-exclusion process was first studied in [21], the Leroux system has been derived as a hydrodynamic limit in [24], and hyperbolic systems have also been studied in [25].

Here we consider a system of two ZRPs. We show that the hydrodynamic equation is a quasilinear parabolic system of the form

1 $$\begin{aligned} \partial _t\varvec{\rho }=\Delta \varvec{\Phi }(\varvec{\rho }),\quad \varvec{\rho }=(\rho _1,\rho _2) :[0,T)\times \mathbbm {T}^d\rightarrow A\subseteq \mathbbm {R}_+^2, \end{aligned}$$

where $\partial _t\varvec{\rho }:=(\partial _t\rho _1,\partial _t\rho _2)$, $\Delta \varvec{\Phi }(\varvec{\rho }):=(\Delta \Phi _1(\varvec{\rho }),\Delta \Phi _2(\varvec{\rho }))$ and $\varvec{\Phi }:A\rightarrow \mathbbm {R}_+^2$ is the mean jump rate of the ZRP at a site $x\in \mathbbm {T}_N^d$ under the product and translation invariant equilibrium state of background density $\varvec{\rho }\in A\subseteq \mathbbm {R}_+^2$. Two-species ZRPs, and the phase transition they exhibit, were first studied in [8]. In condensing ZRPs, the set *A* of admissible background densities $\varvec{\rho }$ is a strict subset of $\mathbbm {R}_+^2$. We call such densities *sub-critical*.

One challenge of ZRPs is that they can exhibit condensation phenomena, where particles congregate at the same site [7, 9, 15]. Even for one-species systems, the hydrodynamic limit of ZRPs experiencing condensation is presently unknown. We consider parameter regimes of two-species systems where condensation can occur, but restrict to *sub-critical initial profiles*, i.e., initial data that take values in the set of sub-critical densities. For one-species ZRPs, the analogous result has been established recently [23] and we extend this argument to the two-species case. Specifically, we apply the relative entropy method of H. T. Yau [28], which requires only the one-block estimate proved in Theorem 3.1, not the full replacement lemma [19, Lemma 5.1.10]. Thus it does not require the equilibrium states of the ZRP to have full exponential moments, a property not satisfied by condensing ZRPs. This extension of [23] is non-trivial, for two reasons. The first difficulty is that the relative entropy method requires the existence of $C^{2+\theta }$ solutions to (1) for some $\theta \in (0,1]$, and that the solution remains in the sub-critical region. By a result of Amann [1] it is know that, when starting from $C^{2+\theta }$ initial data, $C^{1,2+\theta }$ solutions of uniformly parabolic systems exist locally in time, i.e., for small time intervals, and are unique. Thus there exists a unique maximally defined classical solution of the parabolic system (1) taking values in the sub-critical region *A*. So our general result on the hydrodynamic limit is local in time, being valid for the largest time interval for which the unique maximal classical $C^{1,2+\theta }$ solution with values in the sub-critical region exists. This result shows that at least for as long as the unique maximal classical solution to the parabolic system (1) is defined, condensation does not occur. The second difficulty to extend the results for one species [19, 23] is that the phase space $\mathbbm {R}_+^2$ is now more complicated, and the one-dimensional arguments used in [19, 23] do not extend directly. In particular, a novel argument is required to extend [19, Lemma 6.1.10]; see Lemma 4.5 and its proof. Specifically, we employ a characterisation of the domain of a convex function via the recession function of its Legendre transform. This characterisation of the domain of convex functions is of interest in its own right in the context of two-species ZRPs. For example, it immediately yields a parametrisation of the boundary of the domain of the partition function via the recession function of the thermodynamic entropy.

Intuitively, condensation means on the level of the governing hydrodynamic limit PDE the formation of singularities where the mass concentrates. For scalar equations, the formation of such singularities can be ruled out by a maximum principle. For systems, however, in general maximum principles do not hold. In this article, we mainly rely on an existence theory for local $C^{1,2+\theta }$ classical solutions established by Amann and focus on proving the local in time hydrodynamic limit. However, for a particular example, the so-called *species-blind process*, we are able to establish a maximum principle and $C^{1+\theta ,2+\theta }$ regularity for the hydrodynamic equation. This allows us to obtain that $C^{1+\theta ,2+\theta }$ solutions exist and remain in the sub-critical region for all times. So in this particular case, the result on the hydrodynamic limit is global in time.

Maximum principles are more complicated for non-linear parabolic systems, since one has to determine the shape of the *invariant region* in which the solution will have to remain [6, 27], while in the scalar case the invariant region is just an interval. For the species-blind process we find that the invariant region of the hydrodynamic equation coincides with the sub-critical region of the ZRP. This is not surprising since the species-blind process is obtained from a one-species ZRP by colouring particles in two colours, say black and white. The dynamics is the usual ZRP dynamics but at each time of a jump from a site *x*, we choose the colour of particle to move with the probabilities given by the ratios of the number of black particles and white particles at *x* to the total number of particles at *x*, ignoring the colour. It would still be interesting to study the class of parabolic systems arising from two-species ZRPs in order to determine the largest class of ZRPs that their sub-critical region is an invariant region of the hydrodynamic limit. This would then provide a way to find the invariant region of the associated parabolic systems by calculating the phase diagram of the underlying ZRP. The study of the system of PDEs arising from the ZRP is a different topic and outside of the scope of this article, which mainly focuses on the passage from the microscopic to the macroscopic level by applying the relative entropy method.

### Plan of the Paper

The paper is organised as follows. In Sect. 2 we collect some preliminary material on two-species ZRPs and describe the particular case of the species-blind ZRP. Section 3 contains the statements of the main results, and in Sect. 4 we give the proofs.

## The Particle Model

We briefly give the definition of two-species ZRPs as Markov jump processes (Sect. A.1.2 in [19]) and their equilibrium states. Main references on this preliminary material are [12, 13]. We take the discrete *d*-dimensional *N*-torus $\mathbbm {T}_N^d$ as underlying lattice. Each particle interacts only with particles in the same lattice site through a function $\varvec{g}=(g_1,g_2) :\mathbbm {N}_0^2\rightarrow \mathbbm {R}_+^2$. Here $g_i(\varvec{k})$ is the jump rate of species of type *i* from any site that contains $\varvec{k}\in \mathbbm {N}_0^2$ particles, i.e., $k_i$ particles of type *i*, for $i=1,2$. We impose the natural condition

2 $$\begin{aligned} g_i(k)=0\quad \text {iff}\quad k_i=0,\qquad \varvec{k}=(k_1,k_2)\in \mathbbm {N}_0^2 \end{aligned}$$

and require

3 $$\begin{aligned} \Vert \partial _ig_i\Vert _\infty :=\sup _{\varvec{k}\in \mathbbm {N}_0^2}|g_i(\varvec{k} +\varvec{e}_i)-g_i(\varvec{k})|<+\infty , \end{aligned}$$

where $\varvec{e}_i=(\delta _{ij})_{j=1,2}$, $i=1,2$, are the unit vectors in $\mathbbm {R}^2$. Note that setting $\varvec{g}^*:=\Vert \partial _1g_1\Vert _\infty \vee \Vert \partial _2g_2\Vert _\infty <+\infty$, we have by (2) and (3) that

4 $$\begin{aligned} |\varvec{g}(\varvec{k})|_p\le \varvec{g}^*|\varvec{k}|_p,\text { for every }\varvec{k}\in \mathbbm {N}_0^2, \end{aligned}$$

where $|\cdot |_p$ denotes the $\ell _p$-norm in $\mathbbm {R}^2$, $p\in [1,+\infty ]$.

The state space of a two-species ZRP consists of all configurations $\varvec{\eta }=(\eta _1,\eta _2) :\mathbbm {T}_N^d\rightarrow \mathbbm {N}_0^2$, so that $\eta _i(x)$ is the number of *i*-type particles at site *x*, for $i=1,2$. For any measurable space *M* we denote by $\mathbbm {P}(M)$ the set of all probability measures on *M*. We write $p\in \mathbbm {P}(\mathbbm {Z}^d)$ for the nearest neighbour (n.n.) elementary step distribution given by

$$\begin{aligned} p(x):=\frac{1}{2d}\sum _{j=1}^d\mathbbm {1}_{\{-e_j,e_j\}}(x),\quad x\in \mathbbm {Z}^d, \end{aligned}$$

and by $p_N\in \mathbbm {P}(\mathbbm {T}_N^d)$ its projection on $\mathbbm {T}_N^d$ given by $p_N(x) := p(x + N\mathbbm {Z}^d)$. Also, given a configuration $\varvec{\eta }\in \mathbbm {M}_N^{d;2}:=(\mathbbm {N}_0^2)^{\mathbbm {T}_N^d}$, we will denote by $\varvec{\eta }^{i;x;y}$, $i = 1,2$, the configuration resulting from $\varvec{\eta }$ by moving a type-*i* particle from *x* to *y*. (If $\eta _i(x) = 0$, then we set $\varvec{\eta }^{i;x;y} = \varvec{\eta }$.) The *two-species n.n. symmetric ZRP with jump rate* $\varvec{g}$ *on the discrete torus* $\mathbbm {T}_N^d:= \{0,1,\ldots ,N-1\}^d$ is the unique Markov jump process on the Skorohod space $D(\mathbbm {R}_+;\mathbbm {M}_N^{d;2})$ of càdlàg paths characterised by the formal generator

5 $$\begin{aligned} L^Nf(\varvec{\eta })=\sum _{i=1,2}\sum _{x,y\in \mathbbm {T}_N^d}\{f(\varvec{\eta }^{i:x,y}) -f(\varvec{\eta })\}g_i(\varvec{\eta }(x))p_N(y-x). \end{aligned}$$

We will denote by $(P_t^N)_{t\ge 0}$ the transition semigroup of the n.n. symmetric ZRP. The communication classes of the stochastic dynamics defined by the generator above are the hyperplanes

$$\begin{aligned} \mathbbm {M}_{N,\varvec{K}}^{d;2}:=\Big \{\varvec{\eta }\in \mathbbm {M}_N^{d;2} \bigm |\sum _{x\in \mathbbm {T}_N^d}\varvec{\eta }(x)=\varvec{K}\Big \} \end{aligned}$$

consisting of a fixed number of particles of each species. Since each set $\mathbbm {M}_{N,K}^{d;2}$ is finite, for each $(N,\varvec{K})\in \mathbbm {N}\times \mathbbm {N}_0^2$ there exists a unique equilibrium distribution $\nu _{N,\varvec{K}}$ supported on $\mathbbm {M}_{N,\varvec{K}}^{d;2}$. The family $\{\nu _{N,\varvec{K}}\}_{(N,\varvec{K})\in \mathbbm {N}\times \mathbbm {N}_0^2}$ is the so-called *canonical ensemble*. However, as proved in [12, Theorem 4.1], in order to have product and translation invariant equilibrium distributions, it is necessary and sufficient that the following compatibility relations for the component functions of two-species jump rates hold,

6 $$\begin{aligned} g_1(\varvec{k})g_2(\varvec{k}-\varvec{e}_1) = g_1(\varvec{k}-\varvec{e}_2)g_2(\varvec{k}),\quad \text { for all }\varvec{k}\in \mathbbm {N}_0^2\text { with }k_1, k_2\ge 1. \end{aligned}$$

Note that due to the compatibility relations (6) any two-species local jump rate $\varvec{g}$ is uniquely determined by $g_1$ and the restriction of $g_2$ to the set $\{0\}\times \mathbbm {N}_0$, since by induction for any $\varvec{k}\in \mathbbm {N}_0^2$

$$\begin{aligned} g_2(\varvec{k})=g_2(0,k_2)\prod _{i=0}^{k_1-1}\frac{g_1(\varvec{k}-i\varvec{e}_1)}{g_1(\varvec{k}-i\varvec{e}_1-\varvec{e}_2)}. \end{aligned}$$

An *increasing path* $\varvec{\gamma }$ *(from* 0) *to* $\varvec{k}\in \mathbbm {N}_0^2$ is any path $\varvec{\gamma }:\{0,\ldots ,k_1+k_2\}\rightarrow \mathbbm {N}_0^2$ such that $\varvec{\gamma }(0) = 0$, $\varvec{\gamma }(k_1 + k_2)=\varvec{k}$ and $\varvec{\gamma }(\ell ) = \varvec{\gamma }(\ell -1)+\varvec{e}_{i_\ell }$ for some $i_\ell \in \{1,2\}$ for all $\ell = 1,\ldots ,k_1+k_2$. For any increasing path $\gamma$ to $\varvec{k}\in \mathbbm {N}_0^2$, the factorial of $\varvec{g}$ along $\varvec{\gamma }$ is defined as

$$\begin{aligned} \varvec{g}!(\varvec{k};\varvec{\gamma }) =\prod _{\ell =1}^{k_1+k_2}g_{i_\ell }(\varvec{\gamma }(\ell )) \end{aligned}$$

for $\varvec{k}\ne 0$; we set $\varvec{g}!(\cdot ) := 1$ if $\varvec{k}=0$. A two-species local jump rate function $\varvec{g}$ that satisfies (6) yields a well-defined function $\varvec{g}!:\mathbbm {N}_0^2\rightarrow (0,\infty )$ by the formula

$$\begin{aligned} \varvec{g}!(\varvec{k}) = \varvec{g}!(\varvec{k};\varvec{\gamma })\quad \text { for some increasing path }\varvec{\gamma }\text { to }\varvec{k}. \end{aligned}$$

For instance

$$\begin{aligned} \varvec{g}!(\varvec{k})&= g_1(1,0)\cdot \ldots \cdot g_1(k_1,0)\cdot g_2(k_1, 1)\cdot \ldots \cdot g_2(k_1,k_2)\\&= g_2(0,1)\cdot \ldots \cdot g_2(0,k_2)\cdot g_1(1,k_2)\cdot \ldots \cdot g_1(k_1,k_2). \end{aligned}$$

According to [12, Theorem 4.1], using the multi-index notation $\varvec{\varphi }^{\varvec{k}}:=\varphi _1^{k_1}\varphi _2^{k_2}$, with $\varvec{\varphi },\varvec{k}\in \mathbbm {R}_+^2$, for two-species symmetric n.n. ZRP satisfying (6), the common one-site marginal $\bar{\nu }_{\varvec{\varphi }}^1$ of the product and translation invariant equilibrium states $\bar{\nu }_{\varvec{\varphi }}^N$ is given by the formula

$$\begin{aligned} \bar{\nu }_{\varvec{\varphi }}^1(\varvec{k})=\frac{1}{Z(\varvec{\varphi })}\frac{\varvec{\varphi }^{\varvec{k}}}{\varvec{g}!(\varvec{k})},\quad \varvec{k}\in \mathbbm {N}_0^2, \end{aligned}$$

for all $\varvec{\varphi }\in \mathbbm {R}_+^2$ such that the series

7 $$\begin{aligned} Z(\varvec{\varphi }):=\sum _{\varvec{k}\in \mathbbm {N}_0^2}\frac{\varvec{\varphi }^{\varvec{k}}}{\varvec{g}!(\varvec{k})} \end{aligned}$$

converges. The function $Z:\mathbbm {R}_+^2\rightarrow [1,+\infty ]$ defined in (7) is called the *partition function*. The main convexity property of *Z* is that the function $\mathcal Z:=Z\circ \exp :\mathbbm {R}^2\rightarrow (1,+\infty ]$ is strictly logarithmically convex where $\exp (\varvec{\mu }) := e^{\varvec{\mu }}:=(e^{\mu _1},e^{\mu _2})$. This can be seen by applying Hölder’s inequality to the functions $\varvec{k}\mapsto e^{\langle \varvec{\mu },\varvec{k}\rangle }$, $\varvec{k}\mapsto e^{\langle \varvec{\nu },\varvec{k}\rangle }$ with respect to the $\sigma$-finite measure $\lambda$ on $\mathbbm {N}_0^2$ given by $\lambda ({\varvec{k}}):=\frac{1}{\varvec{g}!(\varvec{k})}$ and with the pair of conjugate exponents $p=\frac{1}{1-t}$, $q=\frac{1}{t}$ for $t\in (0,1)$ and $\varvec{\mu },\varvec{\nu }\in \mathbbm {R}^2$ such that $\varvec{\mu }\ne \varvec{\nu }$, which yields

$$\begin{aligned} \mathcal Z\big ((1-t)\varvec{\mu }+t\varvec{\nu }\big )=\int e^{(1-t)\langle \varvec{\mu },\varvec{k}\rangle }e^{t\langle \varvec{\nu },\varvec{k}\rangle } d\lambda (\varvec{k})<\mathcal Z(\varvec{\mu })^{(1-t)}\mathcal Z(\varvec{\nu })^t. \end{aligned}$$

Here and in what follows $\langle \varvec{\mu },\varvec{k}\rangle =\mu _1k_1+\mu _2k_2$ denotes the Euclidean inner product of two vectors $\varvec{k},\varvec{\mu }\in \mathbbm {R}_+^2$. We denote by $\mathcal D_Z:=\{\varvec{\varphi }\in \mathbbm {R}_+^2\bigm |Z(\varvec{\varphi })<+\infty \}$ the *proper domain* of Z, which is a complete, i.e., $[0,\varvec{\varphi }]:=[0,\varphi _1]\times [0,\varphi _2]\subseteq \mathcal D_Z$ for all $\varvec{\varphi }\in \mathcal D_Z$, and logarithmically convex set, that, is the set $\mathcal D_\mathcal Z=\log (\mathcal D_Z\cap (0,\infty )^2):=\{\log \varvec{\varphi }:=(\log \varphi _1, \log \varphi _2)\,\big |\,\varvec{\varphi }\in \mathcal D_Z\cap (0,\infty )^2\}$ is convex. The partition function is $C^\infty$ in $\mathcal D_Z^o$ and continuous from below on $\mathcal D_Z$, i.e., for all $\varvec{\varphi }\in \mathcal D_Z$, $\varepsilon >0$ there exists $\delta >0$ such that $|Z(\varvec{\varphi })-Z(\varvec{\psi })|<\varepsilon$ for all $\varvec{\psi }\in D(0,\delta )\cap [0,\varvec{\varphi }]$. Here $D(0,\delta )$ denotes the Euclidean open ball of radius $\delta$ with centre 0 in $\mathbbm {R}_+^2$, i.e., $D(0,\delta )=\big \{\varvec{\varphi }\in \mathbbm {R}_+^2\bigm ||\varvec{\varphi }|_2<\delta \big \}$.

The family of the product and translation invariant equilibrium states is the family $\{\bar{\nu }^N_{\varvec{\varphi }}\}_{\varvec{\varphi }\in \mathcal D_Z}$. This family is usually referred to as the *grand canonical ensemble (GCE)*. In order to ensure that $\mathcal D_Z$ is not trivial, i.e., that $\mathcal D_Z$ contains a neighbourhood of zero in $\mathbbm {R}_+^2$, we must impose the following condition in the definition of two-species local jump rate functions:

8 $$\begin{aligned} \qquad \varphi _{*;1}:=\liminf _{|\varvec{k}|_1\rightarrow +\infty }{\varvec{g}! (\varvec{k})}^{\frac{1}{|\varvec{k}|_1}} > 0. \end{aligned}$$

A two-species local jump rate $\varvec{g}$ satisfies (8) iff $\mathcal D_Z$ contains a neighbourhood of 0 in $\mathbbm {R}_+^2$. In what follows, we consider only two-species local jump rates that satisfy (6) and (8).

It is convenient to have a parametrisation of the GCE by the density. This is done via the density function $\varvec{R} = (R_1,R_2):\mathcal D_Z\rightarrow [0,+\infty ]^2$ defined by

$$\begin{aligned} \varvec{R}(\varvec{\varphi })=\int _{\mathbbm {M}_N^{d;2}}\varvec{\eta }(0) d\bar{\nu }_{\varvec{\varphi }}^N=\Big (\int k_1d\bar{\nu }_{\varvec{\varphi }}^1,\int k_2d\bar{\nu }_{\varvec{\varphi }}^1\Big ). \end{aligned}$$

The *proper domain* of $\varvec{R}$ is the set $\mathcal D_{\varvec{R}}:=\left\{ \varvec{\varphi }\in D_Z\bigm |\varvec{R}(\varvec{\varphi })\in \mathbbm {R}_+^2\right\}$ and by differentiation of bivariate power-series, we have that

9 $$\begin{aligned} \varvec{R}(\varvec{\varphi })=\varvec{\varphi }\cdot \nabla (\log Z)(\varvec{\varphi })\quad \text { on the set }\mathcal D_{\varvec{R}}^o=\mathcal D_Z^o, \end{aligned}$$

where $\varvec{\varphi }\cdot \varvec{\psi }:=(\varphi _1\psi _1,\varphi _2\psi _2)$ denotes the pointwise product of two vectors $\varvec{\varphi },\varvec{\psi }\in \mathbbm {R}_+^2$. Furthermore, this formula extends to the set $\mathcal D_Z\cap \partial \mathcal D_Z$ if we interpret the directional derivatives $\partial _i(\log Z)\in [0,+\infty ]$ as derivatives from the left. With the conventions $\log 0=-\infty$ and $e^{-\infty }=0$ the densities $\varvec{\rho }\in \varvec{R}(\mathcal D_{\varvec{R}})$ can also be parametrised via the chemical potential by the function $\varvec{\mathcal {R}}:=\varvec{R}\circ \exp :\mathcal D_{\varvec{\mathcal {R}}}\rightarrow \varvec{R}(\mathcal D_{\varvec{R}})$, where $\mathcal D_{\varvec{\mathcal {R}}}=\log (\mathcal D_{\varvec{R}}):=\{\log \varvec{\varphi }\in [-\infty ,+\infty )^2|\varvec{\varphi }\in \mathcal D_{\varvec{R}}\}$. For the parametrisation via the chemical potentials $\varvec{\mathcal {R}}(\varvec{\mu })=\nabla (\log \mathcal Z)(\varvec{\mu })$ for all $\varvec{\mu }\in \mathcal D_{\varvec{\mathcal {R}}}^o\cap (-\infty ,+\infty )^2$, where $\mathcal Z=Z\circ \exp$.

The density function $\varvec{R}:\varvec{R}(\mathcal D_{\varvec{R}})\rightarrow \mathcal D_{\varvec{R}}$ is invertible. Indeed, it is straightforward to check (e.g., see [13, (4.10)]) that for all $\varvec{\varphi }\in \mathcal D_{\varvec{R}}^o\cap (0,+\infty )^2$,

$$\begin{aligned} D\varvec{R}(\varvec{\varphi })=D\varvec{\mathcal {R}}(\log \varvec{\varphi }) \begin{pmatrix} \frac{1}{\varphi _1}&{}0\\ 0&{}\frac{1}{\varphi _2} \end{pmatrix}={\mathrm{Cov}}(\bar{\nu }_{\varvec{\varphi }}^1) \begin{pmatrix} \frac{1}{\varphi _1}&{}0\\ 0&{}\frac{1}{\varphi _2} \end{pmatrix}, \end{aligned}$$

where ${\mathrm{Cov}}(\bar{\nu }_{\varvec{\varphi }}^1)$ denotes the covariance matrix

$$\begin{aligned} {\mathrm{Cov}}(\bar{\nu }_{\varvec{\varphi }}^1)_{ij} =\int k_ik_jd\bar{\nu }_{\varvec{\varphi }}^1-\int k_i\bar{\nu }_{\varvec{\varphi }}^1\int k_j\bar{\nu }_{\varvec{\varphi }}^1,\quad i,j=1,2. \end{aligned}$$

This implies that $D\varvec{R}(\varvec{\varphi })$ is diagonisable with strictly positive eigenvalues for all $\varvec{\varphi }\in \mathcal D_{\varvec{R}}^o\cap (0,+\infty )^2$. Furthermore,

10 $$\begin{aligned} \partial _1R_1(\varvec{\varphi })\wedge \partial _2R_2(\varvec{\varphi })>0 \text{ for } \text{ all } \varvec{\varphi }\in \mathcal D_{\varvec{R}}^o \end{aligned}$$

and for $\varvec{\varphi }\in \mathcal D_{\varvec{R}}^o$ such that $\varphi _1\varphi _2=0$, the matrix $D\varvec{R}(\varvec{\varphi })$ is triangular, and thus invertible. Therefore the density function $\varvec{R}:\mathcal D_{\varvec{R}}^o\rightarrow \varvec{R}(\mathcal D_{\varvec{R}}^o)$ is invertible. The fact that $\varvec{R}$ is invertible on all of its domain follows by [13, Proposition 2.3], according to which for every $\varvec{\rho }\in (0,\infty )^2$ there exists a unique maximiser $\bar{\varvec{\Phi }}(\varvec{\rho })\in \mathcal D_{\varvec{R}}\cap (0,\infty )^2$ for the thermodynamic entropy

11 $$\begin{aligned} S(\varvec{\rho }):= \sup _{\varvec{\varphi }\in \mathcal D_Z\cap (0,\infty )^2}\{\langle \varvec{\rho }, \log \varvec{\varphi }\rangle -\log Z(\varvec{\varphi })\}=\langle \varvec{\rho },\log \bar{\varvec{\Phi }}(\varvec{\rho })\rangle -\log Z\big (\bar{\varvec{\Phi }}(\varvec{\rho })\big ). \end{aligned}$$

Obviously, for $\varvec{\rho }=0$ the supremum is attained at $\varvec{\varphi }=0$ (with the convention $0\cdot (-\infty )=0$). Furthermore, since *Z* is non-decreasing with respect to each variable separately, for any $\varvec{\rho }\in \mathbbm {R}_+^2\setminus \{0\}$ with $\rho _1\rho _2=0$, say $\rho _2=0$, the maximisation problem (11) is reduced to the corresponding maximisation problem for one of the one-species jump rate $\hat{g}_1(k):=g_1(k,0)$, $k\in \mathbbm {N}_0$ and the supremum is attained at $\bar{\varvec{\Phi }}(\rho _1,0)=(\hat{\Phi }_1(\rho _1\wedge \hat{\rho }_{c,1}),0)$, where $\hat{\Phi }_1$, $\hat{\rho }_{c,1}$ are the mean jump rate and critical density of the one-species jump rate $\hat{g}_1$ (see [12, Sect. 5.2.1] for the one-species case). Thus for any $\varvec{\rho }\in \mathbbm {R}_+^2$ there exists a unique maximiser $\bar{\varvec{\Phi }}(\varvec{\rho })\in \mathcal D_{\varvec{R}}$ for the thermodynamic entropy $S(\varvec{\rho })$. As in [13, Proposition 2.3] the function $\varvec{\Phi }:\mathbbm {R}_+^2\rightarrow \mathcal D_{\varvec{R}}$ is continuous, $\varvec{\Phi }:=\bar{\varvec{\Phi }}|_{\varvec{R}(\mathcal D_{\varvec{R}})}=\varvec{R}^{-1}$ is the inverse of $\varvec{R}:\mathcal D_{\varvec{R}}\rightarrow \varvec{R}(\mathcal D_{\varvec{R}})$ and

$$\begin{aligned} \bar{\varvec{\Phi }}\big (\mathbbm {R}_+^2\setminus \varvec{R}(\mathcal D_{\varvec{R}})\big ) =\mathcal D_{\varvec{R}}\cap \partial \mathcal D_{\varvec{R}}. \end{aligned}$$

Furthermore $\varvec{R}(\mathcal D_{\varvec{R}})$ is closed in $\mathbbm {R}_+^2$ and $\partial \varvec{R}(\mathcal D_{\varvec{R}})=\varvec{R}(\mathcal D_{\varvec{R}}\cap \partial \mathcal D_{\varvec{R}})$. According to this result $\bar{\varvec{\Phi }}:\mathbbm {R}_+^2\rightarrow \mathcal D_{\varvec{R}}$ is a left inverse for $\varvec{R}$, i.e., $\bar{\varvec{\Phi }}\circ \varvec{R} = \varvec{\Phi }\circ \varvec{R} = \mathbbm {id}_{\mathcal D_{\varvec{R}}}$ and the function

$$\begin{aligned} {\varvec{R}}_c := \varvec{R}\circ \bar{\varvec{\Phi }}:\mathbbm {R}_+^2\rightarrow \varvec{R}(\mathcal D_{\varvec{R}}) \end{aligned}$$

is a continuous projection on $\varvec{R}(\mathcal D_{\varvec{R}})$ with $\varvec{R}_c|_{\varvec{R}(\mathcal D_{\varvec{R}})} = \mathbbm {id}_{\varvec{R}(\mathcal D_{\varvec{R}})}$, satisfying

$$\begin{aligned} \varvec{R}_c\big (\mathbbm {R}_+^2\setminus \varvec{R}(\mathcal D_{\varvec{R}})\big ) = \varvec{R}\big (\mathcal D_{\varvec{R}}\cap \partial \mathcal D_{\varvec{R}}\big ) = \partial \varvec{R}(\mathcal D_{\varvec{R}}). \end{aligned}$$

In particular, $\varvec{R}:\mathcal D_{\varvec{R}}\rightarrow \varvec{R}(\mathcal D_{\varvec{R}})$ is a homeomorphism and $\varvec{R}(\mathcal D_{\varvec{R}})^o=\varvec{R}(\mathcal D_{\varvec{R}}^o)$.

Note that the thermodynamic entropy coincides with the Legendre transform of the convex *thermodynamic pressure* $\log \mathcal Z:\mathbbm {R}^2\rightarrow (0,+\infty ]$, that is,

12 $$\begin{aligned} S(\varvec{\rho })=(\log \mathcal Z)^*(\varvec{\rho })=\sup _{\varvec{\mu }\in \mathbbm {R}^2} \big \{\langle \varvec{\rho },\varvec{\mu }\rangle -\log \mathcal Z(\varvec{\mu })\big \}. \end{aligned}$$

Since $\nabla (\log \mathcal Z)=\varvec{\mathcal {R}}=\varvec{R}\circ \exp$, it follows by the formula for the derivative of the Legendre transforms that for all $\varvec{\rho }\in (0,\infty )^2\cap \varvec{R}(\mathcal D_{\varvec{R}}^o)$ the supremum in (12) is attained at

$$\begin{aligned} \nabla S(\varvec{\rho })=(\nabla \log \mathcal Z)^{-1}(\varvec{\rho })=\varvec{\mathcal {R}}^{-1}(\varvec{\rho }) =\log \varvec{\Phi }(\varvec{\rho }). \end{aligned}$$

Since *S* is convex the matrix $D^2S(\varvec{\rho })=D(\log \varvec{\Phi })(\varvec{\rho })$ is symmetric and strictly positive definite for all $\varvec{\rho }\in \varvec{R}(\mathcal D_{\varvec{R}}^o)\cap (0,+\infty )^2$. The symmetry of $D^2S(\varvec{\rho })$, $\varvec{\rho }\in \varvec{R}(\mathcal D_{\varvec{R}}^o)\cap (0,+\infty )^2$, implies the relations

13 $$\begin{aligned} \Phi _2(\varvec{\rho })\partial _2\Phi _1(\varvec{\rho })=\Phi _1(\varvec{\rho }) \partial _1\Phi _2(\varvec{\rho }), \end{aligned}$$

which extend to $\varvec{\rho }\in \varvec{R}(\mathcal D_{\varvec{R}}^o)$ because $R_i(\varvec{\varphi })=0$ if and only if $\varphi _i=0$, $i=1,2$ and $D\varvec{R}(\varvec{\varphi })$ is triangular for $\varvec{\varphi }\in \mathcal D_{\varvec{R}}^o$ with $\varphi _1\varphi _2=0$. Equation (13) can be seen as the macroscopic analogue of the compatibility relations (6).

Using the inverse $\varvec{\Phi }$ of $\varvec{R}$ on $\varvec{R}(\mathcal D_{\varvec{R}})$, we can parametrise the grand canonical measures $\bar{\nu }^N_{\varvec{\varphi }}$, $\varvec{\varphi }\in \mathcal D_{\varvec{R}}$, that have finite density via

14 $$\begin{aligned} \nu _{\varvec{\rho }}^N:=\bar{\nu }_{{\varvec{\Phi }}(\varvec{\rho })}^N, \quad \varvec{\rho }\in \varvec{R}(\mathcal D_{\varvec{R}}), \end{aligned}$$

so that they are parametrised by their density. We will denote by $\nu ^\infty _{\varvec{\rho }}:=\bigotimes _{x\in \mathbbm {Z}^d}\nu ^1_{\varvec{\rho }}$, $\varvec{\rho }\in \varvec{R}(\mathcal D_{\varvec{R}})$, the product measures on the configuration space $\mathbbm {M}_\infty ^{d;2}:=(\mathbbm {N}_0^2)^{\mathbbm {Z}^d}$ over the infinite lattice $\mathbbm {Z}^d$. The *logarithmic moment-generating function* $\Lambda _{\varvec{\rho }}:=\Lambda _{\nu _{\varvec{\rho }}^1}:\mathbbm {R}^2 \rightarrow (-\infty ,+\infty ]$ of the one-site marginal $\nu _{\varvec{\rho }}^1$, $\varvec{\rho }\in \mathcal D_{\varvec{R}}$, is defined by

15 $$\begin{aligned} \Lambda _{\varvec{\rho }}^1(\varvec{\lambda }):=\log \int e^{\langle \varvec{\lambda },\varvec{k}\rangle }d\nu _{\varvec{\rho }}^1(\varvec{k}) =\log \frac{Z(e^{\varvec{\lambda }}\cdot \varvec{\Phi }(\varvec{\rho }))}{Z(\varvec{\Phi }(\varvec{\rho }))}. \end{aligned}$$

Consequently, the product and translation invariant equilibrium states have some exponential moments for all $\varvec{\rho }\in \varvec{R}(\mathcal D_{\varvec{R}}^o)$. They have full exponential moments iff $\mathcal D_Z=\mathbbm {R}_+^2$.

It is easy to verify that $\varvec{\Phi }(\varvec{\rho })$ has a probabilistic interpretation as the one-site mean jump rate with respect to the product and translation invariant equilibrium state of density $\varvec{\rho }\in \varvec{R}(\mathcal D_{\varvec{R}})$, that is

$$\begin{aligned} \varvec{\Phi }(\varvec{\rho })=\int \varvec{g}(\varvec{\eta }(0))d\nu _{\varvec{\rho }}^N, \quad \varvec{\rho }\in \varvec{R}(\mathcal D_{\varvec{R}}). \end{aligned}$$

Since $\bar{\varvec{\Phi }}=\varvec{\Phi }\circ \varvec{R}_c$, it follows by (4) that for all $\varvec{\rho }\in \mathbbm {R}_+^2$

16 $$\begin{aligned} \big |\bar{\varvec{\Phi }}(\varvec{\rho })\big |_1\le \int \big |\varvec{g}(\varvec{\eta }(0)) \big |_1d\nu _{\varvec{R}_c(\varvec{\rho })}^N\le \varvec{g}^*\int |\varvec{\eta } (0)|_1d\nu _{\varvec{R}_c(\varvec{\rho })}^N =\varvec{g}^*|\varvec{R}_c(\varvec{\rho })|_1\le \varvec{g}^*|\varvec{\rho }|_1. \end{aligned}$$

One says that *the* 2*-species ZRP is condensing* when $\varvec{R}(\mathcal D_{\varvec{R}})\ne \mathbbm {R}_+^2$, in which case there exist densities $\varvec{\rho }\in \mathbbm {R}_+^2$ for which there is no grand canonical equilibrium state of density $\varvec{\rho }$. Since $\varvec{R}(\mathcal D_{\varvec{R}})$ is non-empty and closed in $\mathbbm {R}_+^2$ it follows that $\varvec{R}(\mathcal D_{\varvec{R}})\ne \mathbbm {R}_+^2$ if and only if $\partial \varvec{R}(\mathcal D_{\varvec{R}})\ne \emptyset$, and thus condensation occurs precisely when $\mathcal D_{\varvec{R}}\cap \partial \mathcal D_{\varvec{R}}\ne \emptyset$. By [13, Theorem 3.3] it follows that $\varvec{R}_c(\varvec{\rho })\le \varvec{\rho }$, that is, $R_{c,i}(\varvec{\rho }):=R_i(\bar{\varvec{\Phi }}(\varvec{\rho }))\le \rho _i$, $i=1,2$, for all $\varvec{\rho }\in \mathbbm {R}^2$. One says that *condensation of the* *i* *-th species*, $i=1,2$, *occurs at the density* $\varvec{\rho }\in \mathbbm {R}_+^2$ if $R_{c,i}(\varvec{\rho }) < \rho _i$. All cases are possible, that is, at a given density $\varvec{\rho }\in \mathbbm {R}_+^2$ no condensation, condensation of exactly one species and condensation of both species simultaneously can occur. These cases induce an obvious partition of the phase space $\mathbbm {R}_+^2$.

As proved in [13], the extension $\bar{\varvec{\Phi }}$ is the correct one for the equivalence of ensembles in the sense that $\varvec{R}_c$ gives the correct limiting background density in the thermodynamic limit. In the case of condensation, i.e., when $\varvec{R}(\mathcal D_{\varvec{R}})\ne \mathbbm {R}_+^2$, some additional assumption must be imposed on the jump rate $\varvec{g}$ to ensure that for each $\varvec{\varphi }\in \mathcal D_{\varvec{R}}\cap \partial \mathcal D_{\varvec{R}}$, the one-site marginal $\bar{\nu }_{\varvec{\varphi }}^1$ has heavy tails in the direction normal to the set $\log (\mathcal D_{\varvec{R}}\cap \partial \mathcal D_{\varvec{R}}):=\{\log \varvec{\varphi } |\varvec{\varphi }\in \mathcal D_{\varvec{R}}\cap \partial \mathcal D_{\varvec{R}}\}$ at $\varvec{\mu }:=\log \varvec{\varphi }$. Denoting by $n_{\varvec{\varphi }}$ the normal to $\log (\mathcal D_{\varvec{R}}\cap \partial \mathcal D_{\varvec{R}})$ at $\log \varvec{\varphi }$ (where $n_{(\varphi _1,0)}=\varvec{e}_1$, $n_{(0,\varphi _2)}=\varvec{e}_2$), this means that

17 $$\begin{aligned} \lim _{\begin{array}{c} |\varvec{k}_n|_2\rightarrow +\infty \\ {\varvec{k}_n}/{|\varvec{k}_n|_2}\rightarrow n_{\varvec{\varphi }} \end{array}}\frac{1}{|\varvec{k}_n|_2}\log \bar{\nu }_{\varvec{\varphi }}^1(\varvec{k}_n)=0. \end{aligned}$$

In case $\partial \mathcal D_{\varvec{R}}$ is not differentiable at $\varvec{\varphi }$, (17) is required to hold for the two limiting normal vectors $n_{\varvec{\varphi }}^+$, $n_{\varvec{\varphi }}^-$ at $\log \varvec{\varphi }$. As has been proven in [13, Lemma 3.5], a condition on the jump rate $\varvec{g}$ that guarantees the critical equilibrium states have heavy tails in the direction normal to the logarithm of the boundary is the *regularity of its tails*, in the sense that for any direction $\varvec{\upsilon }\in S^1_+:=S^1\cap \mathbbm {R}_+^2$,

18 $$\begin{aligned} \varphi _{c;2}(\varvec{\upsilon }):=\liminf _{\begin{array}{c} |\varvec{k}|_2\rightarrow +\infty \\ {\varvec{k}}/{|\varvec{k}|_2}\rightarrow \varvec{\upsilon } \end{array}}\varvec{g}!(\varvec{k})^\frac{1}{|\varvec{k}|_2}\in (0,\infty ) \end{aligned}$$

exists as limit and $\varphi _{c;2} :S^1_+\rightarrow (0,\infty )$ is a continuous function of the direction $\varvec{\upsilon }\in S^1_+$. Note that instead of the exponent $p=2$, we could have used any $p\in [1,+\infty ]$, replacing the Euclidean sphere $S^1_+$ with the sphere $S^1_{p,+}:=\{\varvec{x}\in \mathbbm {R}_+^2 \bigm | |\varvec{x}|_p=1\}$ with respect to the $\ell _p$-norm on $\mathbbm {R}_+^2$. According to the equivalence of ensembles [13, Theorem 3.1], if the jump rate has regular tails when $\varvec{R}(\mathcal D_{\varvec{R}})\ne \mathbbm {R}_+^2$, then for all $\varvec{\rho }\in \mathbbm {R}_+^2$

19 $$\begin{aligned} \lim _{\begin{array}{c} N,|\varvec{K}|\rightarrow +\infty \\ \varvec{K}/N^d\rightarrow \varvec{\rho } \end{array}} \frac{1}{N^d}\mathcal H\left( \nu _{N,\varvec{K}}|\nu _{\varvec{R}_c(\varvec{\rho })}^N\right) =0. \end{aligned}$$

Here $\mathcal H(\mu |\nu )$ denotes the *relative entropy* between two probability measures $\mu ,\nu$,

$$\begin{aligned} \mathcal H(\mu |\nu ):= {\left\{ \begin{array}{ll} \int \!\frac{d\mu }{d\nu }\log \frac{d\mu }{d\nu }d\nu \quad &{}\text {if }\mu \ll \nu \\ +\infty \quad &{}\text {otherwise} \end{array}\right. }. \end{aligned}$$

The translation invariance of canonical and grand canonical ensembles and the super-additivity of the relative entropy imply convergence for any finite set $F\subseteq \mathbbm {Z}^d$, i.e.,

$$\begin{aligned} \lim _{\begin{array}{c} N,|\varvec{K}|\rightarrow +\infty \\ \varvec{K}/N^d\rightarrow \varvec{\rho } \end{array}} \mathcal H\left( \nu _{N,\varvec{K}}^F|\nu _{\varvec{R}_c(\varvec{\rho })}^{N,F}\right) =0 \end{aligned}$$

where $\nu _{N,\varvec{K}}^F:=p_{F*}\nu _{N,\varvec{K}}$, $\nu _{\varvec{R}_c(\varvec{\rho })}^{N,F}:=p_{F*}\nu _{\varvec{R}_c(\varvec{\rho })}^N$ are the push-forwards via the natural projection $p_F:\mathbbm {M}_N^{d;2}\rightarrow (\mathbbm {N}_0^2)^F$ and $\mathbbm {T}_N^d$ is considered embedded in $\mathbbm {Z}^d$. In turn this implies that $\nu _{N,\varvec{K}}$ (considered embedded in the larger space $\mathbbm {M}_\infty ^{d;2}$) converges as $\varvec{K}/N^d\rightarrow \varvec{\rho }$ to $\nu _{\varvec{R}_c(\varvec{\rho })}^\infty$ weakly with respect to bounded *cylinder functions* $f:\mathbbm {M}_\infty ^{d;2}\rightarrow \mathbbm {R}$, that is, such that they depend on a finite number of coordinates.

Finally, we briefly recall the notions of local equilibrium and hydrodynamic limits and refer to [19] for more details. We say that a sequence of probability measures $\{\mu ^N\in \mathbbm {P}(\mathbbm {M}_N^{d;2})\}$ is an *entropy-local equilibrium of profile* $\varvec{\rho }\in C(\mathbbm {T}^d;\varvec{R}(\mathcal D_{\varvec{R}}))$ if

20 $$\begin{aligned} \limsup _{N\rightarrow +\infty }\frac{1}{N^d}\mathcal H\left( \mu ^N|\nu _{\varvec{\rho }(\cdot )}^N\right) =0. \end{aligned}$$

Here $\nu _{\varvec{\rho }(\cdot )}^N:=\bigotimes _{x\in \mathbbm {T}_N^d}\nu _{\varvec{\rho }(x/N)}^1$ is the *product measure with slowly varying parameter associated to the profile* $\varvec{\rho }\in C(\mathbbm {T}^d;\varvec{R}(\mathcal D_{\varvec{R}}))$. Given any cylinder function $f:\mathbbm {M}_N^{d;2}\rightarrow \mathbbm {R}$, we set $\widetilde{f}(\varvec{\rho }):=\int \! fd\nu _{\varvec{R}_c(\varvec{\rho })}^N$, $\varvec{\rho }\in \mathbbm {R}_+^2$. By a simple adaptation of [19, Corollary 6.1.3], if $\{\mu ^N\}$ is an entropy-local equilibrium of profile $\varvec{\rho }\in C(\mathbbm {T}^d;\varvec{R}(\mathcal D_{\varvec{R}}))$, then

21 $$\begin{aligned} \lim _{N\rightarrow +\infty }\mathbbm {E}_{\mu ^N}\Big |\frac{1}{N^d}\sum _{x\in \mathbbm {T}_N^d}H \Big (\frac{x}{N}\Big )\tau _xf(\varvec{\eta }) -\int _{\mathbbm {T}^d}H(u)\widetilde{f}\big (\varvec{\rho }(u)\big )du\Big |=0 \end{aligned}$$

for all $H\in C(\mathbbm {T}^d)$ and all bounded cylinder functions $f:\mathbbm {M}_N^{d;2}\rightarrow \mathbbm {R}$, that is, $\mu ^N$ is a *weak local equilibrium of profile* $\varvec{\rho }\in C(\mathbbm {T}^d;\varvec{R}(\mathcal D_{\varvec{R}}))$.

The *hydrodynamic limit (in the diffusive timescale* $t\mapsto tN^2$) of the n.n. two-species ZRP is an evolutionary PDE, such that entropy-local equilibria are *conserved along its solutions (in the diffusive time-scale)* in the following sense: If we start the process from an entropy local equilibrium $\mu _0^N\in \mathbbm {P}(\mathbbm {M}_N^{d;2})$, $N\in \mathbbm {N}$, of some sufficiently regular initial profile $\varvec{\rho }_0:\mathbbm {T}^d\rightarrow \mathbbm {R}_+^2$ at time $t=0$ and if there exists a sufficiently regular solution $\varvec{\rho }$ of the hydrodynamic equation on $[0,T)\times \mathbbm {T}^d$ starting from $\varvec{\rho }_0$, then $\mu _t^N:=\mu _0^NP_{tN^2}^N$ is an entropy-local equilibrium of profile $\varvec{\rho }(t,\cdot )$ for each $t\in [0,T)$.

The main goal of this article is to apply the relative entropy method of H.T. Yau in order to prove the hydrodynamic limit of condensing two-species ZRPs that start from an initial entropy-local equilibrium $\{\mu _0^N\}$ of sub-critical and strictly positive profile $\varvec{\rho }_0\in C(\mathbbm {T}^d;\varvec{R}(\mathcal D_{\varvec{R}}^o)\cap (0,\infty )^2)$, which is stated as Theorem 3.2 below. A main ingredient in the proof of the hydrodynamic limit is the one-block estimate which is stated as Theorem 3.1. The relative entropy method also requires the existence of a $C_{\mathrm{loc}}^{1,2+\theta }$ classical solution $\varvec{\rho }:[0,T)\times \mathbbm {T}^d\rightarrow \varvec{R}(\mathcal D_{\varvec{R}}^o)\cap (0,+\infty )^2$ for the hydrodynamic limit and applies the Taylor expansion for $C^{2+\theta }$ functions to the function $\varvec{\Phi }(\varvec{\rho }_t)$ of the solution $\varvec{\rho }_t$ at each time $t>0$ (see (45)) in order to estimate the entropy production $\partial _t\mathcal H(\mu _t^N|\nu _{\varvec{\rho }_t(\cdot )}^N)$. The sub-criticality of the solution $\varvec{\rho }$, i.e., that $\varvec{\rho }([0,T)\times \mathbbm {T}^d)\subseteq \varvec{R}(\mathcal D_{\varvec{R}}^o)$, is used in Lemma 4.2 and to obtain the bound (52), which is essential in the application of Lemma 4.5. The sub-criticality of the solution $\varvec{\rho }$ is also required for the application of the large deviations Lemma 4.4. Together with the $C^{2+\theta }$ regularity of $\varvec{\rho }_t$ for each $t\ge 0$ it is the main assumption on the solution $\varvec{\rho }$. Furthermore, in the Taylor expansion the quantities $\Phi _i(\varvec{\rho }_t)$, $i=1,2$, appear in the denominator, so we have to assume that the solution $\varvec{\rho }$ is coordinate-wise strictly positive.

As already mentioned in the introduction the expected hydrodynamic limit of the two-species ZRP with product measures is a quasilinear parabolic system of the form (1), which in divergence form is given by

22 $$\begin{aligned} \partial _t\varvec{\rho }={\mathrm{div}}\varvec{\mathcal A}_{\varvec{\Phi }}(\varvec{\rho },\nabla \varvec{\rho }). \end{aligned}$$

Here the divergence with respect to the spatial parameter is applied coordinate-wise, and $\nabla \varvec{\rho }(t,u):=(\nabla \rho _1(t,u),\nabla \rho _2(t,u))\in \mathbbm {R}^{2\times d}$ is the gradient of $\varvec{\rho }$ with respect to the spatial variable $u\in \mathbbm {T}^d$. Furthermore, $\varvec{\mathcal A}_{\varvec{\Phi }}=(A_{\varvec{\Phi }}^1,\mathcal A_{\varvec{\Phi }}^2):\varvec{R} (\mathcal D_{\varvec{R}}^o)\times \mathbbm {R}^{2\times d}\rightarrow \mathbbm {R}^{2\times d}$ is the function given by

$$\begin{aligned} \varvec{\mathcal A}_{\varvec{\Phi }}(\varvec{\rho },\varvec{V})=D\varvec{\Phi }(\varvec{\rho })\varvec{V}, \end{aligned}$$

that is,

$$\begin{aligned} \partial _t\rho _i={\mathrm{div}}\mathcal A^i_{\varvec{\Phi }}(\varvec{\rho },\nabla \varvec{\rho })={\mathrm{div}}\big (\nabla \Phi _i(\varvec{\rho })\nabla \varvec{\rho }\big ) =\Delta \Phi _i(\varvec{\rho }),\quad i=1,2. \end{aligned}$$

Structural properties of the *mobility matrix* $D\varvec{\Phi }:\varvec{R}(\mathcal D_{\varvec{R}}^o)\rightarrow \mathbbm {R}^{2\times 2}$ can be inferred by the properties of $D\varvec{R}$. For example, for all $\varvec{\rho }\in \varvec{R}(\mathcal D_{\varvec{R}}^o)\cap (0,+\infty )^2$,

$$\begin{aligned} D\varvec{\Phi }(\varvec{\rho })= \left( \begin{array}{ll} \Phi _1(\varvec{\rho })&{}\quad 0\\ 0&{}\quad \Phi _2(\varvec{\rho }) \end{array}\right) D^2S(\varvec{\rho }), \end{aligned}$$

where $D^2S(\varvec{\rho })=D(\log \varvec{\Phi })(\varvec{\rho })$ is a strictly positive definite matrix, the second derivative of the thermodynamic entropy, and for all $\varvec{\rho }\in \varvec{R}(\mathcal D_{\varvec{R}}^o)$, the relations (13) hold and

$$\begin{aligned} \partial _1\Phi _1(\varvec{\rho })\wedge \partial _2\Phi _2(\varvec{\rho })>0. \end{aligned}$$

In particular, $D\varvec{\Phi }(\varvec{\rho })$ has positive eigenvalues for all $\varvec{\rho }\in \varvec{R}(\mathcal D_{\varvec{R}}^o)$ and is diagonisable for all $\varvec{\rho }\in \varvec{R}(\mathcal D_{\varvec{R}}^o)\cap (0,+\infty )^2$. For $\varvec{\rho }\in \varvec{R}(\mathcal D_{\varvec{R}}^o)$ with $\rho _1\rho _2=0$, the matrix $D\varvec{\Phi }(\varvec{\rho })$ is triangular. It follows that although $D\varvec{\Phi }(\varvec{\rho })$ is not necessarily symmetric, it is uniformly parabolic away from the critical densities, that is, for any compact $K\subseteq \varvec{R}(\mathcal D_{\varvec{R}}^o)$ the exists $\lambda _K>0$ such that

$$\begin{aligned} \langle \varvec{\xi },D\varvec{\Phi }(\varvec{\rho })\varvec{\xi }\rangle \ge \lambda _K|\varvec{\xi }|^2, \quad \varvec{\rho }\in K,\;\varvec{\xi }\in \mathbbm {R}^2. \end{aligned}$$

By the work [1] of Amann, it is known that for $C^{2+\theta }$ initial data, uniformly parabolic systems in general form have unique maximal $C^{1,2+\theta }$ solutions. Thus, since $D\varvec{\Phi }$ is uniformly parabolic in any compact subset of $\varvec{R}(\mathcal D_{\varvec{R}}^o)$, it follows that for initial data $\varvec{\rho }_0\in C^{2+\theta }(\mathbbm {T}^d;\varvec{R}(\mathcal D_{\varvec{R}}^o)\cap (0,+\infty )^2)$ there exists a unique maximal classical $C_{\mathrm{loc}}^{1,2+\theta }$ solution $\varvec{\rho }:[0,T_{\mathrm{max}})\times \mathbbm {T}^d\rightarrow \varvec{R}(\mathcal D_{\varvec{R}}^o)$ of the parabolic system (1) taking values in the sub-critical region $\varvec{R}(\mathcal D_{\varvec{R}}^o)$. Of course, since we also assume $\varvec{\rho }_0(\mathbbm {T}^d)\subseteq (0,+\infty )^2$, by taking if necessary $T_{\mathrm{max}}$ to be smaller we can assume that $\varvec{\rho }$ takes values in $\varvec{R}(\mathcal D_{\varvec{R}}^o)\cap (0,+\infty )^2$. This establishes the local in time existence of $C^{1,2+\theta }$ sub-critical solutions $\varvec{\rho }$. On the other hand, by the regularity theory of quasilinear uniformly parabolic systems of the form (22), see [5, Theorem 1.2] and the references therein, it is known that weak solutions to such systems exhibit singularities on a closed subset $Q\subseteq [0,T]\times \mathbbm {T}^d$ of zero measure. So we can not simply apply the $C^{2+\theta }$ Taylor expansion on the function $\varvec{\Phi }(\varvec{\rho }_t)$ for all times $t\ge 0$. Furthermore we do not know whether the sub-critical region $\varvec{R}(\mathcal D_{\varvec{R}})$ is an invariant region for the zero range parabolic system (22). These are the two main reasons that force us to rely on Amann’s local in time existence of regular solutions, and prove a local in time version of the hydrodynamic limit. A further study of the PDE system arising as the hydrodynamic limit of a two-species ZRP, although interesting, is outside of the scope of this article, which is the passage from the microscopic to the macroscopic description.

However, in the example of the species-blind ZRP one can take into advantage its relation with a particular one-species ZRP to obtain the global in time existence of $C_{\mathrm{loc}}^{1+\theta ,2+\theta }$ solutions and a type of maximum principle, in which the sub-critical region plays the role of the invariant domain. We prove this in Theorem 3.3.

### The Species-Blind ZRP

We now consider two-species local jump rate functions of the form

23 $$\begin{aligned} g_1(\varvec{k}) = k_1h(k_1 + k_2),\quad g_2(\varvec{k}) = k_2h(k_1 + k_2) \end{aligned}$$

for some function $h:\mathbbm {N}_0\rightarrow \mathbbm {R}_+$ satisfying the non-degeneracy condition $h(k)>0$ for all $k\in \mathbbm {N}$. Any jump rate $\varvec{g}$ of this form satisfies (6) since

$$\begin{aligned} g_1(\varvec{k})g_2(\varvec{k}-\varvec{e}_1) = k_1h(k_1 + k_2)k_2h(k_1 + k_2 - 1) = g_1(\varvec{k} - \varvec{e}_2)g_2(\varvec{k}) \end{aligned}$$

for all $\varvec{k}\in \mathbbm {N}^2$ and the factorial of such a jump rate is given by

$$\begin{aligned} \varvec{g}!(\varvec{k})=1\cdot h(1)\cdot \ldots \cdot k_1\cdot h(k_1)\cdot 1\cdot h(k_1 + 1)\cdot \ldots \cdot k_2\cdot h(k_1 + k_2) =k_1!k_2!h!(k_1 + k_2). \end{aligned}$$

The partition function associated to $\varvec{g}$ is given for $\varvec{\varphi }\in \mathbbm {N}_0^2$ with $\varphi _2 > 0$ by

$$\begin{aligned} Z(\varvec{\varphi })=\sum _{m=0}^\infty \frac{\varphi _2^m}{h!(m)}\sum _{k_1=0}^m \frac{\left( \frac{\varphi _1}{\varphi _2}\right) ^{k_1}}{k_1!(m-k_1)!} =\sum _{m=0}^\infty \frac{\varphi _2^m}{m!h!(m)}\Big (1+\frac{\varphi _1}{\varphi _2} \Big )^m=\hat{Z}(\varphi _1+\varphi _2), \end{aligned}$$

where $\hat{Z}$ is the partition function associated to the one-species rate function $\hat{g}(k):=kh(k)$. So, in what follows, we assume that *h* is of the form $h(k)=\frac{\hat{g}(k)}{k}$, $k\ge 1$, for some one-species local jump rate function $\hat{g}$ with regular tails, i.e., such that the limit inferior $\hat{\varphi }_c:=\liminf _{k\rightarrow +\infty }\hat{g}!(k)^{\frac{1}{k}}>0$ exists as a limit. In this case the function $\varvec{g}$ defined in (23) is a two-species local jump rate. Indeed, the non-degeneracy condition (2) and the Lipschitz condition (3) are easy to verify, as we have seen $\varvec{g}$ satisfies the compatibility condition (6) and obviously $\mathcal D_Z=\{\varvec{\varphi }\in \mathbbm {R}_+^2|\varphi _1+\varphi _2\in \mathcal D_{\hat{Z}}\}$ and $\mathcal D_{\varvec{R}}=\{\varvec{\varphi }\in \mathbbm {R}_+^2|\varphi _1+\varphi _2\in \mathcal D_{\hat{R}}\}$, where $\hat{R}(\varphi ) =\varphi (\log \hat{Z})'(\varphi )$ is the density function associated to the one-species jump rate $\hat{g}$. In particular $\mathcal D_Z\ne \emptyset$ and thus also (8) holds. We will refer to this nearest neighbour two-species ZRP as the *species-blind ZRP corresponding to the* 1*-species jump rate* $\hat{g}$. The density function corresponding to $\varvec{g}$ is given by the formula

$$\begin{aligned} \varvec{R}(\varvec{\varphi })=\Big (\frac{\varphi _1\hat{Z}'(\varphi _1+\varphi _2)}{\hat{Z} (\varphi _1+\varphi _2)}, \frac{\varphi _2\hat{Z}'(\varphi _1+\varphi _2)}{\hat{Z}(\varphi _1+\varphi _2)}\Big ) =\frac{\hat{R}(|\varvec{\varphi }|_1)}{|\varvec{\varphi }|_1}\varvec{\varphi }. \end{aligned}$$

We set $\hat{\Phi }:= \hat{R}^{-1}$ and we will compute the inverse $\varvec{\Phi }$ of $\varvec{R}:\mathcal D_{\varvec{R}}\rightarrow \mathbbm {R}_+^2$ in its image $\varvec{R}(\mathcal D_{\varvec{R}})$. Let $\varvec{\rho }=\varvec{R}(\varvec{\varphi })$. We have to solve the system

24 $$\begin{aligned} \rho _1=\frac{\varphi _1\hat{Z}'(\varphi _1+\varphi _2)}{\hat{Z} (\varphi _1+\varphi _2)},\quad \rho _2 =\frac{\varphi _2\hat{Z}'(\varphi _1+\varphi _2)}{\hat{Z}(\varphi _1+\varphi _2)} \end{aligned}$$

for $(\varphi _1,\varphi _2)$. By adding the two equations we obtain that $\rho _1+\rho _2=\hat{R}(\varphi _1+\varphi _2)$. In particular $\rho _1+\rho _2\in \hat{R}(\mathcal D_{\hat{R}})$ for all $\varvec{\rho }\in \varvec{R}(\mathcal D_{\varvec{R}})$ and $\varphi _1+\varphi _2=\hat{\Phi }(\rho _1+\rho _2)$. Substituting $\varphi _1 + \varphi _2$ with $\hat{\Phi }(\rho _1+\rho _2)$ in both equations in (24), we can solve for $(\varphi _1,\varphi _2)$ to obtain

$$\begin{aligned} \varphi _i=\rho _i\frac{\hat{Z}\big (\hat{\Phi }(\rho _1+\rho _2)\big )}{\hat{Z}' \big (\hat{\Phi }(\rho _1+\rho _2)\big )}= \rho _i\frac{1}{(\log \hat{Z}'\big (\hat{\Phi }(\rho _1+\rho _2)\big )}=\rho _i \frac{\hat{\Phi }(\rho _1+\rho _2)}{\rho _1+\rho _2}, \end{aligned}$$

where the last equality above follows from the identity $\hat{R}(\varphi ) = \varphi (\log Z)'(\varphi )$ for the one-species density and partition functions, since by this identity we have for all $\rho \in (0,\hat{\rho }_c)$ that

$$\begin{aligned} \frac{1}{(\log \hat{Z})'\big (\hat{\Phi }(\rho )\big )}=\frac{\hat{\Phi }(\rho )}{\hat{R}(\hat{\Phi }(\rho ))}=\frac{\hat{\Phi }(\rho )}{\rho }, \end{aligned}$$

where $\hat{\rho }_c$ is the corresponding critical density of the one-species jump rate $\hat{g}$. Consequently, the inverse $\varvec{\Phi }:=\varvec{R}^{-1}:\varvec{R}(\mathcal D_{\varvec{R}})\rightarrow \mathcal D_{\varvec{R}}$ is given by the formula

25 $$\begin{aligned} \varvec{\Phi }(\varvec{\rho })=\Big (\rho _1\frac{\hat{\Phi }(\rho _1+\rho _2)}{\rho _1+\rho _2}, \rho _2\frac{\hat{\Phi }(\rho _1+\rho _2)}{\rho _1+\rho _2}\Big ) =\frac{\hat{\Phi }(|\varvec{\rho }|_1)}{|\varvec{\rho }|_1}\varvec{\rho }. \end{aligned}$$

Thus the expected hydrodynamic equation of the species-blind ZRP is

26 $$\begin{aligned} \partial _t\rho _i=\Delta \Big (\rho _i\frac{\hat{\Phi }(\rho _1+\rho _2)}{\rho _1+\rho _2}\Big ),\quad i=1,2. \end{aligned}$$

Since (26) is the expected hydrodynamic equation of the species-blind ZRP we will refer to it as the *species-blind parabolic system*. A *classical solution to the species-blind parabolic system* is a $C^{1,2}$ function $\varvec{\rho }=(\rho _1,\rho _2):[0,T)\times \mathbbm {T}^d\rightarrow \mathbbm {R}^2$ satisfying (26) with $0\le \rho _1(t,u)+\rho _2(t,u)<\hat{\rho }_c$ for all $(t,u)\in [0,T)\times \mathbbm {T}^d$. Note that for any classical solution $\varvec{\rho }=(\rho _1,\rho _2)$ of the species-blind parabolic system (26) the sum $\rho _1+\rho _2$ satisfies the parabolic equation $\partial _t\rho =\hat{\Phi }(\rho )$ corresponding to the 1-species ZRP of jump rate $\hat{g}(k)=kh(k)$. This remark will allows us to prove the global in time existence of solutions to the species-blind parabolic system. A similar argument was used for two-species simple exclusion processes in [21].

As an example of the nice properties of the species-blind process, we note that the extended mean jump rate $\bar{\varvec{\Phi }}:\mathbbm {R}_+^2\rightarrow \mathcal D_{\varvec{R}}$ of the species-blind process can be computed explicitly and is given by

$$\begin{aligned} \bar{\varvec{\Phi }}(\varvec{\rho })=\frac{\bar{\hat{\Phi }}(|\varvec{\rho }|_1)}{|\varvec{\rho }|_1}\varvec{\rho }, \end{aligned}$$

where $\bar{\hat{\Phi }}(\rho )=\hat{\Phi }(\rho \wedge \hat{\rho }_c)$, $\rho \ge 0$, is the extended mean jump rate of the one-species ZRP with jump rate $\hat{g}$.

## Main Results

A main probabilistic ingredient in the proof of the hydrodynamic limit of ZRPs is the so-called one-block estimate, which is well known under assumptions that exclude condensing ZRPs (e.g., [19, Sect. 5.4]). Our first result is a version of the one-block estimate for condensing ZRPs, i.e., $\varvec{R}(\mathcal D_{\varvec{R}})\ne \mathbbm {R}_+^2$, under the additional assumptions that the local jump rate $\varvec{g}$ is bounded, has a continuous partition function *Z*, and has regular tails in the sense of (18). We note that these extra assumptions in the one-block estimate and the hydrodynamic limit below are not required in the non-condensing case, i.e., when $\varvec{R}(\mathcal D_{\varvec{R}})=\mathbbm {R}_+^2$. In the case that $\varvec{R}(\mathcal D_{\varvec{R}})=\mathbbm {R}_+^2$, but $\mathcal D_Z\ne \mathbbm {R}_+^2$, Theorems 3.1 and 3.2 still hold under the (weaker than boundedness) assumption that $\varvec{g}$ has sub-linear growth at infinity in the sense that

27 $$\begin{aligned} \limsup _{|\varvec{k}|_1\rightarrow +\infty }\frac{|\varvec{g}(\varvec{k})|_1}{|\varvec{k}|_1}=0. \end{aligned}$$

In the case that $\varvec{R}(\mathcal D_{\varvec{R}})=\mathcal D_Z=\mathbbm {R}_+^2$, no extra assumption is required on $\varvec{g}$. Given any (cylinder) function $\varvec{f}:\mathbbm {M}_N^{d;2}\rightarrow \mathbbm {R}^2$ we set

$$\begin{aligned} \varvec{f}^\ell :=\frac{1}{(2\ell +1)^d}\sum _{|x|\le \ell }\tau _x\varvec{f}, \end{aligned}$$

where $\tau _x\varvec{f}(\varvec{\eta }):=\varvec{f}(\tau _x\varvec{\eta })$ and $\tau _x\varvec{\eta }(y):=\varvec{\eta }(x+y)$ for $x,y\in \mathbbm {T}_N^d$.

### Theorem 3.1

(One-block estimate) Suppose that the ZRP is condensing and that the local jump rate $\varvec{g}$ of the ZRP is bounded, has regular tails in the sense of (18) and its partition function *Z* is continuous on $\mathcal D_Z\cap \partial \mathcal D_Z$. Then for any sequence of initial distributions $\mu _0^N\in \mathbbm {P}(\mathbbm {M}_N^{d;2})$ satisfying the $O(N^d)$-entropy assumption, i.e.,

28 $$\begin{aligned} C(\varvec{a}):=\limsup _{N\in \mathbbm {N}}\frac{1}{N^d}\mathcal H\left( \mu _0^N|\nu _{\varvec{a}}^N\right) <+\infty , \end{aligned}$$

for some (and thus for any) $\varvec{a}\in \varvec{R}(\mathcal D_{\varvec{R}}^o)\cap (0,\infty )^2$, it holds that

29 $$\begin{aligned} \lim _{\ell \rightarrow \infty }\limsup _{N\rightarrow \infty }\mathbbm {E}^N\bigg | \int _0^T\frac{1}{N^d}\sum _{x\in \mathbbm {T}_N^d} \Big \langle \varvec{F}\Big (t,\frac{x}{N}\Big ),\varvec{g}(\varvec{\eta }_t(x)) -\bar{\varvec{\Phi }} \big (\varvec{\eta }_t(x)^\ell \big )\Big \rangle dt\bigg |=0 \end{aligned}$$

for all functions $\varvec{F}\in C([0,T]\times \mathbbm {T}^d;\mathbbm {R}^2)$, $T>0$; $\mathbbm {E}^N$ denotes the expectation with respect to the diffusively accelerated law of the ZRP starting from $\mu _0^N\in \mathbbm {P}(\mathbbm {M}_N^{d;2})$ and $\bar{\varvec{\Phi }}$ is the extension of $\varvec{\Phi }$ given by (11).

We note that the extension $\bar{\varvec{\Phi }}$ of the mean jump rate is required in the statement of the one-block estimate, because $\varvec{\eta }_t^\ell$ can be outside the domain of sub-critical densities. This is the correct extension due to the equivalence of ensembles. The proof of this result is given in Sect. 4.1 below.

Next is the general result regarding the hydrodynamic limit of two-species ZRPs. As noted in the introduction, in order to take into account condensing ZRPs, we apply the relative entropy method of H.T. Yau which requires only the one-block estimate and not the full replacement lemma. But this method relies on the existence of sufficiently regular classical solutions of parabolic systems which are known to exist only locally in time, and thus the result is local in time, valid for the time interval that the unique maximal classical solution of (1) established in [1] exists. We denote by $C^{1+a,2+b}([0,T]\times \mathbbm {T}^d)$, $a,b\in [0,1)$, the space of all $C^{1,2}$-functions $f:[0,T]\times \mathbbm {T}^d\rightarrow \mathbbm {R}$ such that $\partial _tf\in C^a([0,T]\times \mathbbm {T}^d)$ is *a*-Hölder continuous and $\partial ^2_{ij}f\in C^b([0,T]\times \mathbbm {T}^d)$ is *b*-Hölder continuous, where $[0,T]\times \mathbbm {T}^d$ is equipped with the parabolic metric *d* given by

$$\begin{aligned} d\big ((t,x),(s,y)\big )=(d_{\mathbbm {T}^d}(x,y)^2+|t-s|)^\frac{1}{2}. \end{aligned}$$

As usual, if $I\subseteq \mathbbm {R}$ is an interval, then we write $C^{1+a,2+b}_{\mathrm{loc}}(I\times \mathbbm {T}^d)$ for the space of all functions *f* such that $f\in C^{1+a,2+b}(J\times \mathbbm {T}^d)$ for any compact sub-interval $J\subseteq I$. This is extended coordinate-wise to vector-valued functions; given a subset $A\subseteq \mathbbm {R}^2$, we denote by $C^{1+a,2+b}_{\mathrm{loc}}(I\times \mathbbm {T}^d;A)$ the subset of $C^{1+a,2+b}_{\mathrm{loc}}(I\times \mathbbm {T}^d;\mathbbm {R}^2)$ consisting of functions taking values in *A*.

### Theorem 3.2

(Hydrodynamic limit) Let $(S_t^N)_{t\ge 0}$ be the transition semigroup of the two-species symmetric n.n. ZRP on the torus $\mathbbm {T}_N^d$, $N\in \mathbbm {N}$, with condensing jump rate $\varvec{g}$ satisfying the assumptions of the one-block estimate above, and let $\varvec{\Phi }$ be the mean jump rate associated to $\varvec{g}$. Let

$$\begin{aligned} \varvec{\rho }\in C^{1,2+\theta }_{\mathrm{loc}}\big ([0,T_{\mathrm{max}})\times \mathbbm {T}^d;\varvec{R}(\mathcal D_{\varvec{R}}^o)\cap (0,\infty )^2\big ) \end{aligned}$$

be the unique maximal solution of the parabolic system (1) with values in the sub-critical region $\varvec{R}(\mathcal D_{\varvec{R}}^o)\cap (0,+\infty )^2$ of strictly positive densities. Then any initial entropy local equilibrium $\mu _0^N\in \mathbbm {P}(\mathbbm {M}_N^{d;2})$ is conserved along the solution $\varvec{\rho }$. In other words, if $\{\mu _0^N\}$ is an entropy-local equilibrium of profile $\varvec{\rho }_0:=\varvec{\rho }(0,\cdot )\in C^{2+\theta }(\mathbbm {T}^d)$ then $\mu _t^N:=S_{tN^2}^N\mu _0^N$, with $N\in \mathbbm {N}$, is an entropy local equilibrium for all $t\in [0,T_{\mathrm{max}})$. In particular, $\{\mu _t^N\}$ satisfies (21) for all $t\in [0,T_{\mathrm{max}})$.

This theorem is proved in Sect. 4.2. We should note that, although the proof of the hydrodynamic limit relies strongly on the assumption that the classical solution $\varvec{\rho }$ takes values in the set $\varvec{R}(\mathcal D_{\varvec{R}}^o)\cap (0,\infty )^2$ for all times $t\ge 0$, and so in particular requires the sequence of initial distributions $\{\mu _0^N\}$ to be an entropy local equilibrium of some sub-critical and strictly positive profile $\varvec{\rho }_0\equiv \varvec{\rho }(0,\cdot )$, the one-block estimate does not require this assumption. It only requires that $\{\mu _0^N\}$ satisfies the $O(N^d)$-entropy assumption, which can hold even for super-critical profiles, having a Dirac mass of order $O(N^d)$ at some site $x\in \mathbbm {T}^d$, e.g., $\mu _0^N(d\varvec{\eta })=\delta _{[\varvec{a} N^d]}(d\varvec{\eta }_{[Nx]})\otimes \bigotimes _{y\ne [Nx]}\nu _{\varvec{\rho }(y/N)}^1(d\varvec{\eta }_y)$ with $\varvec{a}\in (0,\infty )^2$, when $\varvec{R}(\mathcal D_{\varvec{R}})\ne \mathbbm {R}_+^2$.

We note also that the assumption that $\varvec{\rho }([0,T_{\mathrm{max}})\times \mathbbm {T}^d)\subseteq (0,+\infty )^2$ is a technical one, arising from the fact the $\varvec{\Phi }(\varvec{\rho }_t)$ appears in the denominator. If one knew that the region $\mathbbm {R}_+^2$ is strongly invariant for the parabolic system (1) in the sense that $\rho _1\wedge \rho _2$ becomes strictly positive (and sufficiently fast) for the solution $\varvec{\rho }$, then one can replace the assumption $\varvec{\rho }([0,T_{\mathrm{max}})\times \mathbbm {T}^d)\subseteq (0,+\infty )^2$ with the assumption $\varvec{\rho }_0\ge 0$ as in [23, Remark 3.3] for the one-species case. Secondly, if one knew that the region $(0,+\infty )^2$ is invariant for the parabolic system (1), then starting from $C^{2+\theta }$ non-negative initial data $\varvec{\rho }_0:\mathbbm {T}^d\rightarrow \varvec{R}(\mathcal D_{\varvec{R}}^o)$ one could could choose small enough $\varepsilon >0$ such that $\varvec{\rho }_0^\varepsilon (\mathbbm {T}^d)\subseteq \varvec{R}(\mathcal D_{\varvec{R}}^o)\cap (0,+\infty )^2$ where $\rho ^\varepsilon _{0,i}=\rho _{0,i}+\varepsilon$, $i=1,2$, use the result for strictly positive data and try to pass to the limit as $\varepsilon \rightarrow 0$. Since we do not pursue the study of the quasilinear parabolic system (1) and its invariant regions at the macroscopic level in this article, we consider only local solutions which are strictly positive and sub-critical and whose existence is established by Amann [1].

The next result states that, when starting from sufficiently regular subcritical initial profiles, the species-blind system (26) has solutions defined globally in time.

### Theorem 3.3

(Global existence for the species-blind parabolic system) Let $\varvec{\rho }_0 \in C^{2+\theta }(\mathbbm {T}^d;\varvec{R}(\mathcal D_{\varvec{R}}^o)\cap (0,\infty )^2)$, $\theta \in [0,1)$, be an initial profile. Then the species-blind parabolic system (26) has a unique classical solution $\varvec{\rho }:\mathbbm {R}_+\times \mathbbm {T}^d\rightarrow \mathbbm {R}^2$ starting from $\varvec{\rho }_0$ and

$$\begin{aligned} \varvec{\rho }\in C^{1+\theta ,2+\theta }_{\mathrm{loc}}([0,+\infty )\times \mathbbm {T}^d;\varvec{R}(\mathcal D_{\varvec{R}}^o)\cap (0,\infty )^2). \end{aligned}$$

The proof of this Theorem can be found in Subsect. 4.3 and it is obtained by taking into account the fact that the sum $\rho _1+\rho _2$ of the two variables of a solution $\varvec{\rho }=(\rho _1,\rho _2)$ of the species-blind parabolic system is a solution of the scalar parabolic equation $\partial _t(\rho )=\Delta \hat{\Phi }(\rho )$. Here, by using the strong maximum principle for scalar quasilinear parabolic equations and by proving that classical solutions $\varvec{\rho }$ of the species-blind parabolic system do not become negative, we obtain that the the sub-critical region is an invariant region. We believe that $\mathbbm {R}_+^2$ will be an invariant region of the species-blind parabolic system in general. Yet, since we do not study this question in this article, in order to be rigorous we prove it in this particular case. We should add that the arguments used strongly rely on the relation to the PDE of the single species ZRP associated to the species-blind ZRP by “ignoring” the species, and thus do not easily extend to the general case.

As a corollary, we find that the hydrodynamic limit for the species-blind process holds globally in time; Subsect. 4.4 gives the proof.

### Corollary 3.1

Let $(S_t^N)_{t\ge 0}$ be the transition semigroup of the diffusively rescaled species-blind symmetric n.n. ZRP on the torus $\mathbbm {T}_N^d$ corresponding to a one-species jump rate $\hat{g}$ such that $\hat{\varphi }_c:=\liminf _{k\rightarrow +\infty }\hat{g}!(k)^\frac{1}{k}\in (0,+\infty ]$ exists as a limit. Assume further that $\hat{g}$ is bounded if the critical density $\hat{\rho }_c$ of the one-species ZRP is finite. If $\mu _0^N\in \mathbbm {P}(\mathbbm {M}_N^{d;2})$ is an entropy local equilibrium of profile $\varvec{\rho }_0\in C^{2+\theta }(\mathbbm {T}^d;\varvec{R}(\mathcal D_{\varvec{R}}^o)\cap (0,\infty )^2)$, then $\mu _t^N:=\mu _0^NS_t^N$ is an entropy local equilibrium of profile $\varvec{\rho }(t,\cdot )$ for all $t\ge 0$, where $\varvec{\rho }\in C^{1+\theta ,2+\theta }_{\mathrm{loc}}(\mathbbm {R}_+\times \mathbbm {T}^d;\varvec{R}(\mathcal D_{\varvec{R}}^o)\cap (0,\infty )^2)$ is the unique solution to the species-blind parabolic system (26) starting from $\varvec{\rho }_0$.

## Proofs

### Proof of Theorem 3.1

The proof of the one-block estimate follows closely the proof for the one-species case found in [19, Sect. 5.4]. The differences are twofold. In [19, Sect. 5.4], the one-species case is treated, and we extend this result to two species. However, the main difference is that in [19] the non-condensing case is treated, while we cover the condensing case as well. This is shown by applying the equivalence of ensembles (19) as in [23].

The first step in the proof of the one-block estimate is to replace the jump rate $\varvec{g}(\varvec{\eta }(x))$ at the site *x* with the spatial average $\varvec{g}(\varvec{\eta }(x))^\ell$ over a box of size $\ell \in \mathbbm {N}_0$. This is based on the following lemma which is also useful in the proof of Theorem 3.2. The proof is omitted as it is a simple adaptation of the proof for the one-species case [19, Lemma 6.4.1].

#### Lemma 4.1

If the sequence $\{\mu _0^N\}$ of initial distributions satisfies the $O(N^d)$-entropy assumption (28), then

$$\begin{aligned} \int |\varvec{\eta }|_1d\mu _0^N\le O(N^d), \end{aligned}$$

where $$|\varvec{\eta }|_1 := |\varvec{\eta }|_{N,1}:=\sum _{x\in \mathbbm {T}_N^d}|\varvec{\eta }(x)|_1$$.

This lemma, a change of variables and the conservation of the number of particles allow us to replace $\varvec{g}(\varvec{\eta }(x))$ with the spatial average $\varvec{g}(\varvec{\eta }(x))^\ell$ in the statement of the one-block estimate, and thus the one-block estimate is reduced to proving that

30 $$\begin{aligned} \lim _{\ell \rightarrow \infty }\limsup _{N\rightarrow \infty }\int \frac{1}{N^d}\sum _{x\in \mathbbm {T}^d} \tau _xV^\ell d\bar{\mu }_T^N=0, \end{aligned}$$

where $\bar{\mu }_T^N:=\frac{1}{T}\int _0^T\mu _t^Ndt$ and $V^\ell$ is the cylinder function $V^\ell :=|\varvec{g}(\varvec{\eta }(0))^\ell -\bar{\varvec{\Phi }}(\varvec{\eta }(0)^\ell )|_1$.

We establish this identity in a sequence of steps. We first estimate the entropy and the Dirichlet form of the density $\bar{f}_T^N:={d\bar{\mu }_T^N}/{d\nu _{\varvec{\rho }_*}^N}$ of $\bar{\mu }_T^N$ with respect to an equilibrium state of density $\varvec{\rho }_*\in A$. Note that $\bar{f}_T^N=\frac{1}{T}\int f_t^Ndt$, where $f_t^N:={d\mu _t^N}/{d\nu _{\varvec{\rho }_*}^N}$ is the density of the law $\mu _t^N$ of the ZRP at time *t* with respect to the product equilibrium state of density $\varvec{\rho }_*\in A$. By [19, Proposition A.9.1], for any initial probability measure $\mu$ the entropy $H(\mu _t|\nu )$ of the law $\mu _t:=\mu P_t$ of a Markov semigroup $(P_t)_{t\ge 0}$ at time *t* with respect to an equilibrium state $\pi$ of $(P_t)$ is a non-increasing function of time. Here the equilibrium $\pi$ need not be unique or approached by $\mu _t$ as $t\rightarrow +\infty$. Therefore, since $\mu _0^N$ satisfies the $O(N^d)$-entropy assumption, we have for fixed $\varvec{\rho }_*\in A$ that $H(\mu _t^N|\nu _{\varvec{\rho }_*}^N)\le C(\varvec{\rho }_*)N^d$, which, by convexity of the entropy, implies that $H(\bar{\mu }_T^N|\nu _{\varvec{\rho }_*}^N)\le C(\varvec{\rho }_*)N^d$. Furthermore, if $D_N:L^1_+(\nu _{\varvec{\rho }_*}^N)\rightarrow [0,+\infty ]$ denotes the functional defined by $D_N(f)=\mathfrak {D}_N(\sqrt{f})$ where $\mathfrak {D}_N:L^2(\nu _{\varvec{\rho }_*})\rightarrow [0,+\infty ]$ is the Dirichlet form associated to the generator $L_N$,

$$\begin{aligned} \mathfrak {D}_N(f):=-\langle f,L_Nf\rangle _{\nu _{\varvec{\rho }_*}}=-\int fL_Nfd\nu _{\varvec{\rho }_*}, \end{aligned}$$

then by [19, Proposition A.9.2] and the convexity of the functional $D_N$, it follows that $D_N(\bar{f}_T^N)\le \frac{1}{T}\int _0^TD_N(f_t^N)dt\le \frac{C(\varvec{\rho }_*)}{2T}N^{d-2}$. Therefore, if we set $H_N(f):=H(fd\nu _{\varvec{\rho }_*}^N|\nu _{\varvec{\rho }_*}^N)$, in order to prove the one-block estimate, it suffices to prove that for some $\varvec{\rho }_*\in A$

31 $$\begin{aligned} \limsup _{\ell \rightarrow \infty }\limsup _{N\rightarrow \infty }\sup _{\begin{array}{c} H_N(f)\le C_0N^d\\ D_N(f)\le C_0N^{d-2} \end{array}}\int \frac{1}{N^d}\sum _{x\in \mathbbm {T}_N^d}\tau _xV^\ell fd\nu _{\varvec{\rho }_*}^N\le 0,\quad \forall \;C_0>0, \end{aligned}$$

where the supremum is taken among all densities $f\in L^1_+(\nu _{\varvec{\rho }_*}^N)$.

In a second step, following the proof of the one-species case [19, Sect. 5.4] we cut off large densities. Since Lemma 4.1 requires only the $O(N^d)$-entropy assumption, it follows that

32 $$\begin{aligned} \limsup _{N\rightarrow +\infty }\sup _{H_N(f)\le CN^d}\frac{1}{N^d}\int |\varvec{\eta }|_1fd\nu _{\varvec{\rho }_*}^N<+\infty , \text { for every } C>0. \end{aligned}$$

Similarly to the one-species case, under the assumption that $\varvec{g}$ has sublinear growth at infinity in the sense of (27) (which always holds when $\varvec{g}$ is bounded), inequality (32) allows us to cut off large densities, by restricting $V^\ell$ to the set of configurations $\varvec{\eta }$ which satisfy $|\varvec{\eta }^\ell (0)|_1\le C_1$ for some constant $C_1>0$. This way the one-block estimate is reduced to proving that for all constants $C_0,C_1>0$

33 $$\begin{aligned} \lim _{\ell \rightarrow +\infty }\limsup _{N\rightarrow +\infty }\sup _{D_N(f)\le C_0N^{d-2}}\int \frac{1}{N^d}\sum _{x\in \mathbbm {T}_N^d}\tau _xV^\ell \mathbbm {1}_{\{|\varvec{\eta }^\ell (x)|_1\le C_1\}}fd\nu _{\varvec{\rho }_*}^N\le 0. \end{aligned}$$

In a third step, by adapting the steps 2 to 4 of [19, Sect. 5.4.1] to the two-species case, the one-block-estimate is further reduced to showing that for all constants $C_1>0$,

34 $$\begin{aligned} \limsup _{\ell \rightarrow +\infty }\max _{\varvec{K} \bigm | |\varvec{K}|_1\le (2\ell +1)^dC_1}\int V^\ell d\nu _{2\ell +1,\varvec{K}}=0, \end{aligned}$$

where the canonical measure $\nu _{2\ell +1,\varvec{K}}$ is considered as a measure on $\mathbbm {M}_\infty ^d$ by identifying the cube $\Lambda _\ell ^d:=\{x\in \mathbbm {Z}^d\bigm ||x|\le \ell \}\subseteq \mathbbm {Z}^d$ with $\mathbbm {T}_{2\ell +1}^d$.

The final step in the proof of the one-block estimate consists in applying the equivalence of ensembles to prove (34). Since the measure $\nu _{2\ell +1,\varvec{K}}$ is concentrated on configurations with $\varvec{K}$ particles, the integral appearing in (34) is equal to

$$\begin{aligned} \int V^\ell d\nu _{2\ell +1,\varvec{K}}=\int \bigg |\frac{1}{(2\ell +1)^d}\sum _{|x|\le \ell } \varvec{g}\big (\varvec{\xi }(x)\big ) -\bar{\varvec{\Phi }}\Big (\frac{\varvec{K}}{(2\ell +1)^d}\Big ) \bigg |_1d\nu _{2\ell +1,\varvec{K}}. \end{aligned}$$

As in the one-species case, by fixing a positive integer *k* which will tend to infinity after taking the limit as $\ell \rightarrow +\infty$, and decomposing the cube $\Lambda _\ell ^d$ in smaller cubes of side-length $2k+1$, the one-block estimate is reduced to showing that

35 $$\begin{aligned} \lim _{k\rightarrow \infty }\lim _{m\rightarrow \infty }S(m,k)=0, \end{aligned}$$

where *S*(*m*, *k*) denotes the supremum

$$\begin{aligned} S(m,k):=\sup _{\begin{array}{c} \ell \ge m\\ |\varvec{K}|_1\le (2\ell +1)^dC_1 \end{array}} \int \Big |\frac{1}{(2k+1)^d}\sum _{|x|\le k}\varvec{g}\big (\varvec{\xi }(x)\big )- \bar{\varvec{\Phi }}\Big (\frac{\varvec{K}}{(2\ell +1)^d}\Big )\Big |_1d\nu _{2\ell +1,\varvec{K}}. \end{aligned}$$

This is the part of the proof where we need the boundedness and the regularity of the tails (18) of the jump rate $\varvec{g}$ as well as the continuity of the partition function *Z* on $\mathcal D_Z\cap \partial \mathcal D_Z$. For each fixed $(m,k)\in \mathbbm {N}\times \mathbbm {N}$, we pick a sequence $\{(\ell _n^{m,k},\varvec{K}^{m,k}_n)\}_{n\in \mathbbm {N}}$ such that $\ell _n^{m,k}\ge m$ and $|\varvec{K}_n^{m,k}|_1\le (2\ell _n^{m,k}+1)^dC_1$ for all $n\in \mathbbm {N}$ that achieves the supremum, i.e., such that

$$\begin{aligned} S(m,k)=\lim _{n\rightarrow \infty }\int \Big |\frac{1}{(2k+1)^d}\sum _{|x|\le k}\varvec{g}\big (\varvec{\xi }(x)\big )- \bar{\varvec{\Phi }}\Big (\frac{\varvec{K}^{m,k}_n}{(2\ell _n^{m,k}+1)^d}\Big ) \Big |_1d\nu _{2\ell _n^{m,k}+1,\varvec{K}^{m,k}_n}. \end{aligned}$$

Since the sequence $\{\varvec{r}_n^{m,k}\}_{n\in \mathbbm {N}}$ defined by

$$\begin{aligned} \varvec{r}_n^{m,k}:=\frac{\varvec{K}^{m,k}_n}{(2\ell _n^{m,k}+1)^d},\qquad n\in \mathbbm {N}, \end{aligned}$$

is contained in the compact triangular region $B_{|\cdot |_1}(0,C_1):=\{\varvec{r}\in \mathbbm {R}_+^2 \bigm | |\varvec{r}|_1\le C_1\}$, for each fixed $(m,k)\in \mathbbm {N}\times \mathbbm {N}$, we can pick a sequence $\{n_j\}_{j\in \mathbbm {N}} := \{n_j^{m,k}\}$ such that $\varvec{r}^{m,k}_{n_j}$ converges to some $\varvec{r}^{m,k}\in B_{|\cdot |_1}(0,C_1)$ as $j\rightarrow \infty$. Since we assume that $\varvec{g}$ is bounded, it follows by the equivalence of ensembles that

$$\begin{aligned} S(m,k)=\int \Big |\frac{1}{(2k+1)^d}\sum _{|x|\le k}\varvec{g}\big (\varvec{\xi }(x)\big )- \bar{\varvec{\Phi }}\big (\varvec{r}^{m,k}\big )\Big |_1d\nu _{\varvec{R}_c (\varvec{r}^{m,k})}^\infty . \end{aligned}$$

Furthermore, since $|\varvec{R}_c(\varvec{\rho })|_1\le |\varvec{\rho }|_1$, for each fixed $k\in \mathbbm {N}$ the sequence $\{\varvec{\rho }^{m,k}:=\varvec{R}_c(\varvec{r}^{m,k})\}_{m\in \mathbbm {N}}$, is also contained in $B_{|\cdot |_1}(0,C_1)$ and thus we can choose a sequence $\{m_j\}_{j\in \mathbbm {N}}=\{m_j^{(k)}\}$ such that $\{\varvec{\rho }^{m_j,k}\}_{m\in \mathbbm {N}}$ converges to some $\varvec{\rho }^k\in B_{|\cdot |_1}(0,C_1)\cap \varvec{R}(\mathcal D_{\varvec{R}})$. By the continuity assumption on *Z*, the grand canonical ensemble is weakly continuous. By this fact, the continuity of $\varvec{R}_c$ and the identity $\bar{\varvec{\Phi }}=\varvec{\Phi }\circ \varvec{R}_c$,

$$\begin{aligned} \lim _{m\rightarrow \infty }S(m,k)=\int \Big |\frac{1}{(2k+1)^d}\sum _{|x|\le k}\varvec{g}\big (\varvec{\xi }(x)\big )- \varvec{\Phi }\big (\varvec{\rho }^k\big )\Big |_1d\nu _{\varvec{\rho }^k}^\infty . \end{aligned}$$

Therefore

$$\begin{aligned} \limsup _{k\rightarrow +\infty }\lim _{m\rightarrow +\infty }S(m,k)\le \limsup _{k\rightarrow \infty }\sup _{\varvec{\rho }\in \varvec{R}(\mathcal D_{\varvec{R}})}\int \Big |\frac{1}{(2k+1)^d} \sum _{|x|\le k}\varvec{g}\big (\varvec{\eta }(x)\big )- \varvec{\Phi }\big (\varvec{\rho }\big )\Big |_1d\nu _{\varvec{\rho }}^\infty . \end{aligned}$$

The random variables $\varvec{g}\big (\varvec{\eta }(x)\big )$, $x\in \mathbbm {Z}^d$, are uniformly bounded by $\Vert \varvec{g}\Vert _\infty$ and i.i.d. with respect to $\nu _{\varvec{\rho }}^\infty$ for all $\varvec{\rho }\in \varvec{R}(\mathcal D_{\varvec{R}})$ and thus they satisfy the $L^2$-weak law of large numbers uniformly over all parameters $\varvec{\rho }\in \varvec{R}(\mathcal D_{\varvec{R}})$, which shows that the term in the right hand side above is equal to zero. This completes the proof of the one-block estimate and hence the proof of Theorem 3.1. $\square$

### Proof of Theorem 3.2

Let *A* be the interior of the set of all strictly positive sub-critical densities, i.e.,

$$\begin{aligned} A:=\varvec{R}(\mathcal D_{\varvec{R}}^o)\cap (0,\infty )^2, \end{aligned}$$

and let $\varvec{\rho }:[0,T_{\mathrm{max}})\times \mathbbm {T}^d\rightarrow A$ be the maximal classical solution established in [1] of the initial value problem (1) with $\varvec{\rho }(0,\cdot ) := \varvec{\rho }_0\in C^{2+\theta }(\mathbbm {T}^d;A)$. We fix $\varvec{a}\in A$ and denote by $\psi ^{N}_t$ the Radon-Nikodym derivative of $\nu _{\varvec{\rho }_t(\cdot )}^N$ with respect to $\nu _{\varvec{a}}^N$,

$$\begin{aligned} \psi _t^N:=\frac{d\nu _{\varvec{\rho }_t(\cdot )}^N}{d\nu _{\varvec{a}}^N}. \end{aligned}$$

Let $H_N(t):=\mathcal H(\mu _t^N|\nu _{\varvec{\rho }_t(\cdot )}^N)$ be the relative entropy of $\mu _t^N$ with respect to $\nu _{\varvec{\rho }_t(\cdot )}^N$. We have the following upper bound on the entropy production, proved in [19, Lemma 6.1.4],

36 $$\begin{aligned} \partial _tH_N(t)\le \int \frac{1}{\psi _t^N}\big \{N^2L_N^*\psi _t^N-\partial _t \psi _t^N\big \}d\mu _t^N \end{aligned}$$

for every $t\in [0,T_{\mathrm{max}})$, where $L_N^*$ is the adjoint of $L_N$ in $L^2(\nu _{\varvec{a}}^N)$. Denoting by

37 $$\begin{aligned} H(t):=\limsup _{N\rightarrow \infty }\frac{1}{N^d}H_N(t),\quad t\in [0,T_{\mathrm{max}}), \end{aligned}$$

the limiting entropy density, the main step in the application of the relative entropy method is to use this upper bound on $\partial _tH_N(t)$ to get an inequality of the form

38 $$\begin{aligned} H(t)\le H(0)+ \frac{1}{\gamma }\int _0^tH(s)ds \end{aligned}$$

for some constant $\gamma >0$. Since $H(0)=0$ by assumption, this implies by Gronwall’s inequality that $H(t)=0$ for all $t\in [0,T_{\mathrm{max}})$ as required. Of course, in order for Gronwall’s inequality to be applicable, *H* must belong at least in $L^1_{{\mathrm{loc}}}([0,T_{\mathrm{max}})]$. This is the context of the next two lemmas. The first is Remark 6.1.2 in [19] for single-species ZRPs.

#### Lemma 4.2

If $\{\mu _0^N\}$ is an entropy local equilibrium of profile $\varvec{\rho }\in C(\mathbbm {T}^d;\varvec{R}(\mathcal D_{\varvec{R}}^o))$, then $\{\mu _0^N\}$ satisfies the $O(N^d)$-entropy assumption (28).

#### Proof

Indeed, for fixed $\varvec{a}\in A:=\varvec{R}(\mathcal D_{\varvec{R}}^o)\cap (0,\infty )^2$, by the relative entropy inequality [19, Sect. A.1.8]

39 $$\begin{aligned} \mathcal H(\mu _0^N|\nu _{\varvec{a}}^N) \le \Big (1+\frac{1}{\gamma }\Big )\mathcal H(\mu _0^N|\nu _{\varvec{\rho }(\cdot )}^N) +\frac{1}{\gamma }\log \int e^{\gamma \log \frac{d\nu _{\varvec{\rho }(\cdot )}^N}{d\nu _{\varvec{a}}^N}} d\nu _{\varvec{\rho }(\cdot )}^N. \end{aligned}$$

Since $\nu _{\varvec{\rho }(\cdot )}^N$, $\nu _{\varvec{a}}^N$ are product measures, the Radon-Nikodym derivative $\frac{d\nu _{\varvec{\rho }(\cdot )}^N}{d\nu _{\varvec{a}}^N}$ can be computed explicitly. With the notation $\varvec{\Phi }_{\varvec{a}}:=\big (\frac{\Phi _1}{\Phi _1(\varvec{a})},\frac{\Phi _2}{\Phi _2(\varvec{a})}\big )$, $Z_{\varvec{a}}:=\frac{Z\circ \varvec{\Phi }}{Z(\varvec{\Phi }(\varvec{a}))}$

$$\begin{aligned} \int \Big (\frac{d\nu _{\varvec{\rho }(\cdot )}^N}{d\nu _{\varvec{a}}^N}\Big )^\gamma d\nu _{\varvec{\rho }(\cdot )}^N=\prod _{x\in \mathbbm {T}_N^d}\frac{1}{Z_{\varvec{a}}(\varvec{\rho } (x/N))^\gamma }\int e^{\langle \varvec{k},\gamma \log \varvec{\Phi }_{\varvec{a}}(\varvec{\rho }(x/N))\rangle } d\nu _{\varvec{\rho }(x/N)}^1(\varvec{k}). \end{aligned}$$

Since $Z\ge 1$, we have that $\frac{1}{Z_{\varvec{a}}(\varvec{\rho })}=\frac{Z(\varvec{\Phi }(\varvec{a}))}{Z({\varvec{\Phi }}(\varvec{\rho }))}\le Z(\varvec{\Phi }(\varvec{a}))$ and therefore

40 $$\begin{aligned} \frac{1}{\gamma N^d}\log \int \Big (\frac{d\nu _{\varvec{\rho }(\cdot )}^N}{d\nu _{\varvec{a}}^N}\Big )^\gamma d\nu _{\varvec{\rho }(\cdot )}^N&\le Z(\varvec{\Phi }(\varvec{a}))+\frac{1}{\gamma N^d}\sum _{x\in \mathbbm {T}_N^d}\Lambda _{\varvec{\rho }(x/N)} \big (\gamma \log \varvec{\Phi }_{\varvec{a}}\big (\varvec{\rho } \big (x/N\big )\big )\big )\nonumber \\&\le Z(\varvec{\Phi }(\varvec{a}))+\frac{1}{\gamma N^d}\sum _{x\in \mathbbm {T}_N^d}\log Z\big (\varvec{F}_{\varvec{a}}(x/N,\gamma )\big ), \end{aligned}$$

where here $\varvec{F}_{\varvec{a}}:\mathbbm {T}^d\times [0,1]\rightarrow (0,\infty )^2$ is the function given by $\varvec{F}_{\varvec{a}}(u,\gamma )=\frac{\varvec{\Phi }(\varvec{\rho }(u))^{1+\gamma }}{\varvec{\Phi }(\varvec{a})^\gamma }$ and for $\varvec{a}\in \mathbbm {R}_+^2$, $\varvec{b}\in (0,\infty )^2$, $\gamma >0$, we have set $\varvec{a}^\gamma :=(a_1^\gamma ,a_2^\gamma )$ and $\frac{\varvec{a}}{\varvec{b}}:=(\frac{a_1}{b_1},\frac{a_2}{b_2})$. Since $\varvec{\rho }(\mathbbm {T}^d)\subseteq \varvec{R}(\mathcal D_{\varvec{R}}^o)$ by assumption, it follows that $\varvec{\Phi }(\varvec{\rho }(\mathbbm {T}^d))\subseteq \mathcal D_Z^o$. Since $\varvec{F}_{\varvec{a}}$ is uniformly continuous on $\mathbbm {T}^d\times [0,1]$ and satisfies $\lim _{\gamma \rightarrow 0}\varvec{F}_{\varvec{a}}(u,\gamma )=\varvec{\Phi }(\varvec{\rho }(u))$ for all $u\in \mathbbm {T}^d$, it follows that its image is contained in $\mathcal D_Z^o$, i.e., $\{\varvec{F}_{\varvec{a}}(u,\gamma )|u\in \mathbbm {T}^d\}\subseteq \mathcal D_Z^o$ for sufficiently small $\gamma >0$. Then the function $u\mapsto Z(\varvec{F}_{\varvec{a}}(u,\gamma ))$ is well defined and continuous on the torus $\mathbbm {T}^d$, so that its Riemannian sums converge. By (39), (40) and the fact that $\mu _0^N$ is an entropy local equilibrium, this yields that

$$\begin{aligned} C(\varvec{a})\le Z(\varvec{\Phi }(\varvec{a}))+\frac{1}{\gamma }\int _{\mathbbm {T}^d}\log Z\big (\varvec{F}_{\varvec{a}}(u,\gamma ))du<+\infty \end{aligned}$$

for small $\gamma >0$, and the proof of Lemma 4.2 is complete. $\square$

#### Lemma 4.3

Let $\varvec{\rho }:[0,T]\times \mathbbm {T}^d\rightarrow \varvec{R}(\mathcal D_{\varvec{R}}^o)\cap (0,\infty )^2$ be a continuous function and let $\{\mu _0^N\}$ be an entropy local equilibrium with respect to $\varvec{\rho }_0:=\varvec{\rho }(0,\cdot )$. Then the upper entropy $\overline{H} :[0,T]\rightarrow [0,+\infty ]$ defined by

$$\begin{aligned} \overline{H}(t):=\sup _{N\in \mathbbm {N}}\frac{1}{N^d}\mathcal H\left( \mu _t^N|\nu _{\varvec{\rho }_t(\cdot )}^N\right) \end{aligned}$$

belongs to $$L^\infty ([0,T])$$.

#### Proof

By the relative entropy inequality and [19, Proposition A.1.9.1], according to which the function $t\mapsto \mathcal H(\mu _t^N|\nu _{\varvec{a}}^N)$ is non-increasing,

41 $$\begin{aligned} H_N(t)\le \Big (1+\frac{1}{\gamma }\Big )\mathcal H(\mu _0^N|\nu _{\varvec{a}}^N)+\frac{1}{\gamma }\log \int \Big (\frac{d\nu _{\varvec{a}}^N}{d\nu _{\varvec{\rho }_t(\cdot )}^N}\Big )^\gamma d\nu _{\varvec{a}}^N \end{aligned}$$

for all $t\ge 0$ and all $\gamma >0$. Since the proper domain of $Z_{\varvec{a}}$ has interior $\mathcal D_{Z_{\varvec{a}}}^o=\varvec{R}(\mathcal D_{\varvec{R}}^o)$ and since $\varvec{\rho }([0,T]\times \mathbbm {T}^d)\subseteq \varvec{R}(\mathcal D_{\varvec{R}}^o)\cap (0,\infty )^2$, the function $Z_{\varvec{a}}\circ \varvec{\rho }$ is a bounded continuous function on the torus, and therefore, by a computation similar to the one in the proof of Lemma 4.2, we obtain

$$\begin{aligned}&\frac{1}{\gamma N^d}\log \int \Big (\frac{d\nu _{\varvec{a}}^N}{d\nu _{\varvec{\rho }_t(\cdot )}^N}\Big )^\gamma d\nu _{\varvec{a}}^N\\&\quad =\Vert Z_{\varvec{a}}\circ \varvec{\rho }\Vert _{L^\infty ([0,T]\times \mathbbm {T}^d)}+\frac{1}{\gamma N^d}\sum _{x\in \mathbbm {T}_N^d}\Lambda _{\varvec{a}}\Big (\gamma \log \frac{1}{\varvec{\Phi }_{\varvec{a}} (\varvec{\rho }_t(x/N))}\Big ), \end{aligned}$$

where $\Vert Z_{\varvec{a}}\circ \varvec{\rho }\Vert _{L^\infty ([0,T]\times \mathbbm {T}^d)}<+\infty$. For the second term, we have for every $u\in \mathbbm {T}^d$ that

42 $$\begin{aligned} \Lambda _{\varvec{a}}\Big (\gamma \log \frac{1}{\varvec{\Phi }_{\varvec{a}} (\varvec{\rho }_t(u))}\Big ) =\log \Big \{\frac{1}{Z(\varvec{\Phi }(\varvec{a}))} Z\Big (\frac{\varvec{\Phi }(\varvec{a})^{1+\gamma }}{\varvec{\Phi } (\varvec{\rho }_t(u))^{\gamma }}\Big )\Big \}. \end{aligned}$$

Since $\varvec{\Phi }(\varvec{\rho })(\mathbbm {T}^d)\subseteq (0,\infty )^2$ and $\varvec{\Phi }(\varvec{\rho })$ is continuous, there exists $\varvec{\varphi }_0\in \mathcal D_Z$ such that $\varvec{\varphi }_0<\varvec{\Phi }(\varvec{\rho }(t,u))$ for all $(t,u)\in [0,T]\times \mathbbm {T}^d$. Then since *Z* is increasing,

$$\begin{aligned} Z\Big (\frac{\varvec{\Phi }(\varvec{a})^{1+\gamma }}{\varvec{\Phi } (\varvec{\rho }_t(u))^{\gamma }}\Big )\le Z\Big (\frac{\varvec{\Phi }(\varvec{a})^{1+\gamma }}{\varvec{\varphi }_0^\gamma }\Big ), \end{aligned}$$

and since $\varvec{\Phi }(\varvec{a})\in \mathcal D_Z^o$ and $\varvec{\Phi }(\varvec{a})^{1+\gamma }/\varvec{\varphi }_0^\gamma \rightarrow \varvec{\Phi }(\varvec{a})$ as $\gamma \rightarrow 0$, we can choose $\gamma _0>0$ sufficiently small so that $\varvec{\Phi }(\varvec{a})^{1+\gamma }/\varvec{\varphi }_0^\gamma \in \mathcal D_Z^o$ and $Z\big ({\varvec{\Phi }(\varvec{a})^{1+\gamma }}/{\varvec{\varphi }_0^\gamma }\big )\le Z\big (\varvec{\Phi }(\varvec{a})\big )+1$ for all $\gamma <\gamma _0$. Consequently, since by Lemma 4.2 $\{\mu _0^N\}$ satisfies the $O(N^d)$-entropy assumption, by (41) for some constant $C\ge 0$ for all $\gamma <\gamma _0$

$$\begin{aligned} \Vert \overline{H}\Vert _{L^\infty ([0,T])}\le \Big (1+\frac{1}{\gamma }\Big )C+\Vert Z_{\varvec{a}} (\varvec{\rho })\Vert _{L^\infty ([0,T]\times \mathbbm {T}^d)}+\frac{1}{\gamma } \log \frac{Z(\varvec{\Phi }(\varvec{a}))+1}{Z(\varvec{\Phi }(\varvec{a}))}<+\infty , \end{aligned}$$

establishing the claim of Lemma 4.3. $\square$

The bound (36) on the entropy production can be estimated explicitly. Since $\nu _{\varvec{\rho }_t(\cdot )}^N$, $\nu _{\varvec{a}}^N$ are product measures, $\psi _t$ can be computed explicitly. Then by differentiating, using the chain rule, the fact that $\varvec{\rho }$ is a solution of the hydrodynamic equation, the relations $\frac{\varphi _i\partial _iZ(\varvec{\varphi })}{Z(\varvec{\varphi })}=R_i(\varvec{\varphi })$, $i=1,2$ and the relation (13) we obtain

43 $$\begin{aligned} \frac{\partial _t\psi _t^N}{\psi _t^N}=\sum _{x\in \mathbbm {T}_N^d} \Big \langle \frac{\Delta \varvec{\Phi }(\varvec{\rho }_t(x/N))}{\varvec{\Phi }(\varvec{\rho }_t(x/N))}, D\varvec{\Phi }\big (\varvec{\rho }_t(x/N)\big )[\varvec{\eta }(x) -\varvec{\rho }_t(x/N)]\Big \rangle . \end{aligned}$$

We note here that as in the asymmetric case treated in [16], at this point of the application of the relative entropy method for two-species ZRPs one has to use the macroscopic analogue (13) of the compatibility relations (6).

For the other term, by computations of the action of the generator on $\psi _t^N$ similar to the ones for the single-species case in [19],

44 $$\begin{aligned} \frac{L_N^*\psi _t^N}{\psi _t^N}=\sum _{i=1,2}\sum _{x,y\in \mathbbm {T}_N^d} \Big [\frac{\Phi _i\big (\varvec{\rho }_t(y/N)\big )}{\Phi _i\big (\varvec{\rho }_t(x/N)\big )}-1\Big ] \big [g_i\big (\varvec{\eta }(x)\big )-\Phi _i\big (\varvec{\rho }_t(x/N) \big )\big ]p(y-x).\quad \end{aligned}$$

Since $\varvec{\Phi }(\varvec{\rho }_t)$ is $C^{2+\theta }$ for some $\theta >0$ and the n.n. transition probability has mean zero, the Taylor expansion for $C^{2+\theta }$ functions yields (with the renormalisation $p(\mathbbm {Z}^d)=2d$) that

45 $$\begin{aligned} \frac{N^2L_N^*\psi _t^N}{\psi _t^N}=\sum _{x\in \mathbbm {T}_N^d} \Big \langle \frac{\Delta [\varvec{\Phi }(\varvec{\rho }_t)]}{\varvec{\Phi }(\varvec{\rho }_t)} \left( \frac{x}{N}\right) ,\varvec{g}(\varvec{\eta }(x)) -\varvec{\Phi }(\varvec{\rho }_t(x/N))\Big \rangle +r_N(t). \end{aligned}$$

Here, for any $T\in [0,T_{\mathrm{max}})$, the remainder $r_N(t)$ satisfies the bound

$$\begin{aligned} |r_N(t)|\le \frac{C_T\varvec{g}^*}{N^\theta }|\varvec{\eta }|_1 +\frac{C_TM_T}{m_T}N^{d-\theta } \end{aligned}$$

for all $t\in [0,T]$, where $\varvec{g}^*$ is the constant in (4), $C_T=C(d,p,\varvec{\Phi }(\varvec{\rho }),T)\ge 0$ is the constant

$$\begin{aligned} C_T=\sqrt{d}\sup _{0\le t\le T}\Vert D^2[\Phi _1(\varvec{\rho }_t)]\Vert _{C^{\theta }}\vee \Vert D^2[\Phi _2(\varvec{\rho }_t)]\Vert _{C^{\theta }}\sum _{y\in \mathbbm {Z}^d} \Vert y\Vert ^{2+\theta }p(y) \end{aligned}$$

with $\Vert \cdot \Vert _{C^\theta }$ denoting the $\theta$-Hölder seminorm and

$$\begin{aligned} m_T:=\inf _{(t,u)\in [0,T]\times \mathbbm {T}^d}\min _{i=1,2}\Phi _i(\varvec{\rho }_t(u))>0,\qquad M_T:=\sup _{(t,u)\in [0,T]\times \mathbbm {T}^d}|\varvec{\Phi }(\varvec{\rho }(t,u))|_1<+\infty . \end{aligned}$$

By this bound on the remainder and the conservation of the number of particles it follows that for all $t\in [0,T]\subseteq [0,T_{\mathrm{max}})$, $T>0$,

$$\begin{aligned} \frac{1}{N^d}\int _0^t\int r_N(t)d\mu _t^Ndt\le \frac{C_T\varvec{g}^*t}{N^{d+\theta }}\int |\varvec{\eta }|_1d\mu _0^N+\frac{C_TM_Tt}{m_T} \frac{1}{N^\theta }, \end{aligned}$$

which according to Lemma 4.1 shows that

46 $$\begin{aligned} \int _0^t\int r_N(s)d\mu _s^Nds\le o(N^d). \end{aligned}$$

Since the function $\frac{\Delta [\varvec{\Phi }(\varvec{\rho }_t)]}{\varvec{\Phi }(\varvec{\rho }_t)}$ is in $C_{\mathrm{loc}}([0,T_{\mathrm{max}})\times \mathbbm {T}^d)$, a change of variables shows that

47 $$\begin{aligned} \int _0^t\int \sum _{x\in \mathbbm {T}_N^d}\Big \langle \frac{\Delta [\varvec{\Phi }(\varvec{\rho }_s)]}{\varvec{\Phi }(\varvec{\rho }_s)} \left( \frac{x}{N}\right) ,\varvec{\eta }(x)-\varvec{\eta }^\ell (x)\Big \rangle d\mu _s^N ds=o(N^d) \end{aligned}$$

for all $t\in [0,T_{\mathrm{max}})$. Integrating (36) in time, using the explicit expressions (43), (45), taking into account (46) and the fact that $\{\mu _0^N\}$ is an entropy local equilibrium (i.e., (20) holds) and using (47) and the one-block estimate, one obtains that for all $t\in (0,T_{\mathrm{max}})$

48 $$\begin{aligned} H_N(t)\le \int _0^t\int \sum _{x\in \mathbbm {T}_N^d} \Big \langle \frac{\Delta [\varvec{\Phi }(\varvec{\rho }_s)]}{\varvec{\Phi } (\varvec{\rho }_s)} \left( \frac{x}{N}\right) ,\varvec{\Psi }\big (\varvec{\rho }_s(x/N), \varvec{\eta }^\ell (x)\big )\Big \rangle d\mu _s^Nds+o_\ell (N^d), \end{aligned}$$

where $\varvec{\Psi }:\varvec{R}(\mathcal D_{\varvec{R}}^o)\times \mathbbm {R}_+^2\rightarrow \mathbbm {R}^2$ is the *quasi-potential*

49 $$\begin{aligned} \varvec{\Psi }(\varvec{\rho },\varvec{\lambda })=\bar{\varvec{\Phi }}(\varvec{\lambda }) -\varvec{\Phi }(\varvec{\rho })-D\varvec{\Phi }(\varvec{\rho })(\varvec{\lambda }-\varvec{\rho }) \end{aligned}$$

and the term $o_\ell (N^d)$ satisfies $o_\ell (N^d)/N^d\rightarrow 0$ as *N* and then $\ell$ tend to infinity. In the definition of the quasi-potential the second variable $\varvec{\lambda }$ is in $\mathbbm {R}_+^2$ since it is to be substituted by the large microscopic averages $\varvec{\eta }^\ell (x)$, $x\in \mathbbm {T}_N^d$. Thus the extension $\bar{\varvec{\Phi }}$ of $\varvec{\Phi }$ must be used in the quasi-potential. To simplify the notation, we set

50 $$\begin{aligned} G_t(u,\varvec{\lambda }):=\left\langle \frac{\Delta [\varvec{\Phi }(\varvec{\rho }_t)]}{\varvec{\Phi }(\varvec{\rho }_t)}(u), \varvec{\Psi }\big (\varvec{\rho }_t(u),\varvec{\lambda }\big )\right\rangle . \end{aligned}$$

By the relative entropy inequality, we have for all $\gamma >0$ and all $0\le s<T_{\mathrm{max}}$ that

$$\begin{aligned} \int \sum _{x\in \mathbbm {T}_N^d}G_s\big (x/N,\varvec{\eta }^\ell (x)\big ) d\mu _s^N\le \frac{1}{\gamma }H_N(s)+\frac{1}{\gamma }\log \int e^{\gamma \sum _{x\in \mathbbm {T}_N^d}G_s(\frac{x}{N},\varvec{\eta }^\ell (x))} d\nu _{\varvec{\rho }_s(\cdot )}^N. \end{aligned}$$

By combining this inequality with the bound (48), dividing by $N^d$ and taking the $\limsup$ as $N\rightarrow \infty$ and then $\ell \rightarrow \infty$, we get

51 $$\begin{aligned} H(t)\le \frac{1}{\gamma }\int _0^tH(s)ds+\limsup _{\ell ,N\rightarrow +\infty }\frac{1}{\gamma N^d}\int _0^t\log \int e^{\gamma \sum _{x\in \mathbbm {T}_N^d}G_s(\frac{x}{N},\varvec{\eta }^\ell (x))} d\nu _{\varvec{\rho }_s(\cdot )}^Nds,\quad \end{aligned}$$

where in order to obtain the term $\int _0^tH(s)ds$ we used Lemma 4.3 to pass the limit inside the integral and $\limsup _{\ell ,N\rightarrow +\infty }$ denotes the $\limsup$ as $N\rightarrow +\infty$ and then $\ell \rightarrow +\infty$.

To complete the proof of Theorem 3.2, it remains to show that for each $t\in [0,T_{\mathrm{max}})$ we can choose $\gamma >0$ small enough so that the rightmost term in (51) vanishes. We begin by noting that the function $G:[0,T_{\mathrm{max}})\times \mathbbm {T}^d\times \mathbbm {R}_+^2\rightarrow \mathbbm {R}$ defined in (50) satisfies

$$\begin{aligned} |G_t(u,\varvec{\lambda })| \le \Big |\frac{\Delta [\varvec{\Phi }(\varvec{\rho }_t)]}{\varvec{\Phi }(\varvec{\rho }_t)} (u)\Big |_\infty \Big \{\varvec{g}^*\big (|\varvec{\lambda }|_1 +|(\varvec{\rho }_t(u))|_1\big )+|D\varvec{\Phi }(\varvec{\rho }_t(u))|_\infty \big (|\varvec{\lambda }|_1+|\varvec{\rho }_t(u)|_1\big )\Big \} \end{aligned}$$

for all $(t,u,\varvec{\lambda })\in [0,T_{\mathrm{max}})\times \mathbbm {T}^d\times \mathbbm {R}_+^2$, which for any $t\in [0,T_{\mathrm{max}})$ yields the inequality

52 $$\begin{aligned} \sup _{(s,u)\in [0,t]\times \mathbbm {T}^d}|G_s(u,\varvec{\lambda })|\le C_t\cdot (1+|\varvec{\lambda }|_1) \quad \text {for all }\varvec{\lambda }\in \mathbbm {R}_+^2 \end{aligned}$$

for some constant $C_t<+\infty$. Since for any $t\in [0,T_{\mathrm{max}})$ we have $\varvec{\rho }([0,t]\times \mathbbm {T}^d)\subseteq \varvec{R}(\mathcal D_{\varvec{R}}^o)$ the set $\varvec{\Phi }(\varvec{\rho })([0,T]\times \mathbbm {T}^d)$ is bounded away from the critical densities $\varvec{\varphi }_c\in \partial \mathcal D_Z$, and thus there exists $\varepsilon >0$ such that

$$\begin{aligned} \sup _{(t,u)\in [0,T]\times \mathbbm {T}^d}\Lambda _{\varvec{\rho }(t,u)}(\varvec{\lambda })< +\infty ,\quad \forall \varvec{\lambda }\in D(0,\varepsilon ), \end{aligned}$$

i.e., $0\in \big (\mathcal D_{\sup _{(t,u)\in [0,T]\times \mathbbm {T}^d}\Lambda _{\varvec{\rho }(t,u)}}\big )^o =\big (\bigcap _{(t,u)\in [0,T]\times \mathbbm {T}^d}\mathcal D_{\Lambda _{\varvec{\rho }(t,u)}}\big )^o$. It follows that by choosing $\gamma _t$ small enough so that $\gamma _tC_t<\varepsilon$, we can pass the limit superior as $N\rightarrow +\infty$ and then $\ell \rightarrow +\infty$ inside the time integral in the rightmost term in (51). Thus in order to complete the proof it remains to show that for each $t\in [0,T_{\mathrm{max}})$ we can choose $\gamma _t>0$ small enough so that

53 $$\begin{aligned} \limsup _{\ell ,N\rightarrow +\infty }\frac{1}{\gamma _t N^d}\log \int e^{\gamma _t\sum _{x\in \mathbbm {T}_N^d}G_t(\frac{x}{N},\varvec{\eta }^\ell (x))}d\nu _{\varvec{\rho }_t(\cdot )}^N\le 0. \end{aligned}$$

The proof of (53) relies on a corollary of the Laplace-Varadhan lemma [4, Sect. 4.3] for the large deviations principle satisfied by the independent family of the occupation variables $\{\varvec{\eta }(x)\}_{x\in \mathbbm {Z}^d}$ with respect to the invariant measure $\nu _{\varvec{\rho }}^\infty$ on the infinite lattice $\mathbbm {Z}^d$ for some $\varvec{\rho }\in A$. Since the one-site marginal $\nu _{\varvec{\rho }}^1$ has some exponential moments for $\varvec{\rho }\in A$, by Cramér’s theorem, the large deviations functional of the family $\{\varvec{\eta }(x)\}_{x\in \mathbbm {Z}^d}$ is given by the Legendre transform $\Lambda _{\varvec{\rho }}^*$ of the logarithmic moment-generating functional $\Lambda _{\varvec{\rho }}$. Note that (15) implies that modulo an affine function depending on $\varvec{\rho }$, the rate functional $\Lambda _{\varvec{\rho }}^*$ coincides with the thermodynamic entropy *S*, that is

54 $$\begin{aligned} \Lambda _{\varvec{\rho }}^*(\varvec{\lambda })=S(\varvec{\lambda })-\big \langle \varvec{\lambda }, \log \varvec{\Phi }(\varvec{\rho })\big \rangle +\log Z\big (\varvec{\Phi }(\varvec{\rho })\big ). \end{aligned}$$

#### Lemma 4.4

Let $\varvec{\rho }:\mathbbm {T}^d\rightarrow \varvec{R}(\mathcal D_{\varvec{R}}^o)$ be a continuous profile and let $G:\mathbbm {T}^d\times \mathbbm {R}_+\rightarrow \mathbbm {R}$ be a continuous function such that

55 $$\begin{aligned} \sup _{u\in \mathbbm {T}^d}|G(u,\varvec{\lambda })|\le C(1+|\varvec{\lambda }|_1)\quad \text {for all }\varvec{\lambda }\in \mathbbm {R}_+^2 \end{aligned}$$

for some constant $C>0$ such that $(2C,2C)\in \big (\bigcap _{u\in \mathbbm {T}^d}\mathcal D_{\Lambda _{\varvec{\rho }(u)}}\big )^o$. Then

$$\begin{aligned} \limsup _{\ell \rightarrow \infty }\limsup _{N\rightarrow \infty }\frac{1}{N^d}\log \int e^{\sum _{x\in \mathbbm {T}_N^d}G(\frac{x}{N},\varvec{\eta }^\ell (x))} d\nu _{\varvec{\rho }(\cdot )}^N\le \int _{\mathbbm {T}^d}\sup _{\varvec{\lambda }\in \mathbbm {R}_+^2}\Big \{G(u,\varvec{\lambda }) -\frac{1}{2}\Lambda _{\varvec{\rho }(u)}^*(\varvec{\lambda })\Big \}du. \end{aligned}$$

We omit the proof of this Lemma as it is a simple adaptation of the corresponding result in the one-species case, [19, Lemma 6.1.10]. By the bound (52) the function $G:[0,T_{\mathrm{max}})\times \mathbbm {T}^d\times \mathbbm {R}_+^2\rightarrow \mathbbm {R}$ defined in (50) satisfies

$$\begin{aligned} \sup _{u\in \mathbbm {T}^d}|G_t(u,\varvec{\lambda })|\le C_t(1+|\varvec{\lambda }|_1) \end{aligned}$$

for each fixed $t\in [0,T_{\mathrm{max}})$. Therefore, if we choose $\gamma _t>0$ small enough so that $2\gamma _t C_t(\varvec{e}_1+\varvec{e}_2)\in \big (\bigcap _{u\in \mathbbm {T}^d}\Lambda _{\varvec{\rho }_t(u)}\big )^o$, then for all $\gamma \in (0,\gamma _t)$ the function $\gamma G_t$ satisfies the assumptions of Lemma 4.4, and thus for $\gamma \in (0,\gamma _t)$ the term in (51) is bounded above by

56 $$\begin{aligned} \int _{\mathbbm {T}^d}\sup _{\varvec{\lambda }\in \mathbbm {R}_+^2}\Big \{\gamma G_t(u,\varvec{\lambda })-\frac{1}{2}\Lambda ^*_{\varvec{\rho }_t(u)}(\varvec{\lambda })\Big \}du. \end{aligned}$$

To complete the application of the relative entropy method, it remains to show that by reducing $\gamma _t>0$, $t\in [0,T_{\mathrm{max}})$, if necessary, this last term is non-positive.

We note that this would follow if we had a bound of the form

57 $$\begin{aligned} B_t:=\sup _{\begin{array}{c} \varvec{\rho }\in K_t \\ \varvec{\lambda }\in \mathbbm {R}_+^2 \end{array}} \frac{|\varvec{\Psi }(\varvec{\rho },\varvec{\lambda })|}{\Lambda _{\varvec{\rho }}^*(\varvec{\lambda })}< +\infty , \end{aligned}$$

where $K_t\subseteq A:=\varvec{R}(\mathcal D_{\varvec{R}}^o)\cap (0,\infty )^2$ is a compact set containing the image $\varvec{\rho }_t(\mathbbm {T}^d)$. Indeed, since $\Lambda _{\varvec{\rho }}^*(\varvec{\lambda })=0$ iff $\varvec{\lambda }=\varvec{\rho }$, in which case $\varvec{\Psi }(\varvec{\rho },\varvec{\lambda })=0$, we would then have that

$$\begin{aligned} |\varvec{\Psi }(\varvec{\rho },\varvec{\lambda })|\le B_t\Lambda _{\varvec{\rho }}^*(\varvec{\lambda }) \quad \text { for all }(\varvec{\rho },\varvec{\lambda })\in K_t\times \mathbbm {R}_+^2, \end{aligned}$$

and so for $\gamma \in (0,\gamma _t)$ we would have

$$\begin{aligned} \gamma |G_t(u,\varvec{\lambda })|\le \gamma \left\| \frac{\Delta [\varvec{\Phi }(\varvec{\rho }_t)]}{\varvec{\Phi }(\varvec{\rho }_t)}\right\| _{L^\infty (\mathbbm {T}^d;\ell ^2_\infty )} B_t\Lambda ^*_{\varvec{\rho }_t(u)}(\varvec{\lambda }) \end{aligned}$$

for all $(u,\varvec{\lambda })\in \mathbbm {T}^d\times \mathbbm {R}_+^2$. Then by choosing $\gamma _t>0$ small enough so that in addition $\gamma _tB_t\left\| {\Delta [\varvec{\Phi }(\varvec{\rho }_t)]}/{\varvec{\Phi }(\varvec{\rho }_t)} \right\| _{L^\infty (\mathbbm {T}^d;\ell ^2_\infty )}<\frac{1}{2}$, it would follow that (56) is non-positive, and the proof would be complete. The bound (57) is proved in Lemma 4.5. Before we proceed with the proof of Lemma 4.5, we recall some facts on recession functions of convex functions.

Given a lower semicontinuous proper convex function $\psi :\mathbbm {R}^d\rightarrow (-\infty ,+\infty ]$ with $0\in \mathcal D_\psi$, its *recession function* $\psi _\infty :\mathbbm {R}^d\rightarrow (-\infty ,+\infty ]$ is defined by

$$\begin{aligned} \psi _\infty (y):=\lim _{t\rightarrow +\infty }\frac{\psi (ty)}{t}=\lim _{t\rightarrow +\infty } \frac{d}{dt}\Big |_+\psi (ty), \end{aligned}$$

where $\frac{d}{dt}|_+$ denotes differentiation from the right. The recession function $\psi _\infty$ is obviously positively 1-homogeneous, $\psi _\infty (\lambda y)=\lambda \psi _\infty (y)$ for all $y\in \mathbbm {R}^d$, $\lambda \ge 0$.

It is well known [22, Theorem 8.5] that if $\psi$ is a proper lower semi-continuous convex function, then so is its recession function. Using the equivalent definition of recession functions via the recession cone of their epigraphs [22, Sect. 8], one can express the recession function by the formula

58 $$\begin{aligned} \psi _\infty (y)=\inf \Big \{\liminf _{k\rightarrow +\infty }\frac{\psi (t_ky_k)}{t_k}\Big |t_k\rightarrow +\infty ,\;y_k\rightarrow y\Big \} \end{aligned}$$

(see [10, (12.7.1)]). Particularly useful in the proof of the following lemma is the characterisation of the interior of the proper domain of a convex function $\psi$ via the recession function of its Legendre transform, as stated in [10, (12.7.3)],

59 $$\begin{aligned} \mathcal D_\psi ^o=\bigcap _{y\ne 0}\big \{x\in \mathbbm {R}^d\bigm |\langle x,y\rangle <(\psi ^*)_\infty (y)\big \}. \end{aligned}$$

Applying (59) to the *thermodynamic pressure* $P:=\log \mathcal Z$, $\mathcal Z:=Z\circ \exp$, we get

60 $$\begin{aligned} \log \big (\mathcal D_Z^o\cap (0,\infty )^2\big )=\mathcal D_\mathcal Z^o =\big \{\varvec{\mu }\in \mathbbm {R}^2\bigm |S_\infty (\varvec{\lambda })>\langle \varvec{\lambda }, \varvec{\mu }\rangle ,\;\forall \varvec{\lambda }\ne 0\big \}. \end{aligned}$$

In other words, $\mathcal D_\mathcal Z^o$ is the intersection of all hyperplanes $\{\varvec{\mu }\in \mathbbm {R}^2|\langle \varvec{\mu },\varvec{\upsilon }\rangle <S_\infty (\varvec{\upsilon })\}$ for $\varvec{\upsilon }\in S^1\cap (0,\infty )^2$. This implies that the function $S^1\cap (0,\infty )^2\ni \varvec{\upsilon }\mapsto S_\infty (\varvec{\upsilon })\varvec{\upsilon }\in \mathbbm {R}^2$ is a parametrisation of the boundary $\partial \mathcal D_\mathcal Z$. This may be compared with [13, (2.14)]. Consequently, the part of the boundary $\partial \mathcal D_Z$ on the strictly positive quadrant is given by the parametrisation $e^{S_\infty (\varvec{\upsilon })\varvec{\upsilon }}$, $\varvec{\upsilon }\in S^1\cap (0,\infty )^2$. Along the two axes $\varphi _1=0$ and $\varphi _2=0$, there is only one-species of particles and the critical fugacities in these directions are fugacities of one species ZRPs.

#### Lemma 4.5

For any compact $K\subseteq A:=\varvec{R}(\mathcal D_{\varvec{R}}^o)\cap (0,\infty )^2$,

61 $$\begin{aligned} \sup _{(\varvec{\rho },\varvec{\lambda })\in K\times \mathbbm {R}_+^2}\frac{|\varvec{\Psi }(\varvec{\rho },\varvec{\lambda })|}{\Lambda _{\varvec{\rho }}^*(\varvec{\lambda })}<+\infty . \end{aligned}$$

#### Proof

For all $(\varvec{\rho },\varvec{\lambda })\in K\times (0,\infty )^2$ we have that $\Lambda ^*(\varvec{\rho },\varvec{\lambda }):=\Lambda ^*_{\varvec{\rho }}(\varvec{\lambda })\ge 0$ and the functions $|\varvec{\Psi }| :K\times (0,\infty )^2\rightarrow \mathbbm {R}_+$ and $\Lambda ^* :K\times (0,\infty )^2\rightarrow \mathbbm {R}_+$ are continuous. Therefore the fraction in the supremum can tend to infinity if the nominator goes to infinity or the denominator goes to zero. Since $\varvec{\Psi } :K\times (0,\infty )^2\rightarrow \mathbbm {R}^2$ is continuous and *K* is compact the nominator can tend to infinity only as $|\varvec{\lambda }|_1\rightarrow +\infty$. In this case $\varvec{\Lambda }_{\varvec{\rho }}^*$ also tends to $+\infty$ as a rate functional with compact level sets. Since $\Lambda _{\varvec{\rho }}^*$ is the rate functional of the i.i.d. occupation variables $\varvec{\eta }(x)$, $x\in \mathbbm {Z}^d$, with common law $\nu _{\varvec{\rho }}^1$ we have that $\Lambda ^*(\varvec{\rho },\varvec{\lambda })=0$ iff $\varvec{\rho }=\varvec{\lambda }$ for the denominator. But obviously for $\varvec{\rho }=\varvec{\lambda }$ we have $\varvec{\Psi }(\varvec{\rho },\varvec{\lambda })=0$, so the nominator vanishes as well. So in order to prove the lemma we have to show that the nominator and the denominator are of the same order as $|\varvec{\rho }-\varvec{\lambda }|\rightarrow 0$ and $|\varvec{\rho }-\varvec{\lambda }|\rightarrow +\infty$.

Motivated by the previous sketch, we choose $\varepsilon >0$ such that $K_\varepsilon :=\overline{K^{(\varepsilon )}}\subseteq A$, where $K^{(\varepsilon )}:=\bigcup _{x\in K}D(x,\varepsilon )$, and for any $M>0$ we separate the region $K\times (0,\infty )^2$ as $K\times (0,\infty )^2=\mathcal E_0^\varepsilon \cup \mathcal E_\varepsilon ^M\cup \mathcal E_M^\infty$, where

$$\begin{aligned} \mathcal E_0^\varepsilon&:=\{(\varvec{\rho },\varvec{\lambda })\in K\times (0,\infty )^2\bigm ||\varvec{\rho }-\varvec{\lambda }|\le \varepsilon \},\\ \mathcal E_\varepsilon ^M&:=\{(\varvec{\rho },\varvec{\lambda })\in K\times (0,\infty )^2\bigm |\varepsilon \le |\varvec{\rho }-\varvec{\lambda }|\le M\},\\ \mathcal E_M^\infty&:=\{(\varvec{\rho },\varvec{\lambda })\in K\times (0,\infty )^2\bigm ||\varvec{\rho }-\varvec{\lambda }|\ge M\}. \end{aligned}$$

We prove the claim on each region individually. Obviously the set $\mathcal E_\varepsilon ^M$ is compact and so since the functions $\varvec{\Psi }$ and $\Lambda ^*$ are jointly continuous, the claim holds on the region $\mathcal E_\varepsilon ^M$.

We turn to the region $\mathcal E_0^\varepsilon$. By its definition, for any $(\varvec{\rho },\varvec{\lambda })\in \mathcal E_0^\varepsilon$ we have that $\varvec{\lambda }\in D(\varvec{\rho },\varepsilon )\subseteq K_\varepsilon \subseteq A$. So, since $D(\varvec{\rho },\varepsilon )$ is convex, for all $(\varvec{\rho },\varvec{\lambda })\in \mathcal E_0^\varepsilon$ the image of the constant speed line segment $\varvec{\gamma }_{\varvec{\rho },\varvec{\lambda }}:[0,1]\rightarrow \mathbbm {R}^2$ from $\varvec{\rho }$ to $\varvec{\lambda }$ is contained in $K_\varepsilon$, i.e.,

62 $$\begin{aligned} \varvec{\gamma }_{\varvec{\rho },\varvec{\lambda }}([0,1])\subseteq K_\varepsilon \quad \text { for all }(\varvec{\rho },\varvec{\lambda })\in \mathcal E_0^\varepsilon . \end{aligned}$$

By the first order Taylor expansion of $\Phi _i$, $i=1,2$ around the point $\varvec{\rho }\in K$,

$$\begin{aligned} \Psi _i(\varvec{\rho },\varvec{\lambda })=\int _0^1(1-t)\big \langle \varvec{\lambda } -\varvec{\rho },D^2\Phi _i(\varvec{\gamma }_{\varvec{\rho },\varvec{\lambda }}(t))(\varvec{\lambda } -\varvec{\rho })\big \rangle dt \quad \text {for all }(\varvec{\rho },\varvec{\lambda })\in \mathcal E_0^\varepsilon . \end{aligned}$$

Since $\varvec{\Phi }$ is smooth on the set *A*, the matrix $D^2\Phi _i(\varvec{\rho })$ is symmetric for all $\varvec{\rho }\in A$. Denoting by $\lambda _{\pm }^i(\varvec{\rho })$ the real eigenvalues of $D^2\Phi _i(\varvec{\rho })$ we have

$$\begin{aligned} \lambda _-^i(\varvec{\gamma }_{\varvec{\rho },\varvec{\lambda }}(t))|\varvec{\lambda } -\varvec{\rho }|^2\le \langle \varvec{\lambda }-\varvec{\rho },D^2 \Phi _i(\varvec{\gamma }_{\varvec{\rho },\varvec{\lambda }}(t))(\varvec{\lambda }-\varvec{\rho }) \big \rangle \le \lambda _+^i(\varvec{\gamma }_{\varvec{\rho },\varvec{\lambda }}(t)) |\varvec{\lambda }-\varvec{\rho }|^2. \end{aligned}$$

Furthermore, by the continuity of the eigenvalues $\lambda ^i_\pm$ as functions of $\varvec{\rho }\in A$,

$$\begin{aligned} A^i:=\sup _{\varvec{\rho }\in K_\varepsilon }|\lambda ^i_-|\vee |\lambda ^i_+|(\varvec{\rho })<+\infty . \end{aligned}$$

So by (62), we have that $|\lambda ^i_-|\vee |\lambda ^i_+|(\varvec{\gamma }_{\varvec{\rho },\varvec{\lambda }}(t))\le A^i$ for all $(t,\varvec{\rho },\varvec{\lambda })\in [0,1]\times \mathcal {E}_0^\varepsilon$ and thus

$$\begin{aligned} |\Psi _i(\varvec{\rho },\varvec{\lambda })|\le \frac{A^i}{2}|\varvec{\lambda } -\varvec{\rho }|^2,\quad i=1,2. \end{aligned}$$

For the denominator in (61), we note that the rate functional $\Lambda _{\varvec{\rho }}^*$ is $C^1$ on $(0,\infty )^2$ and $C^2$ on *A* with

$$\begin{aligned} \nabla \Lambda _{\varvec{\rho }}^*(\varvec{\lambda }) =\log \frac{\bar{\varvec{\Phi }}(\varvec{\lambda })}{\varvec{\Phi }(\varvec{\rho })}, \quad \varvec{\lambda }\in (0,\infty )^2, \end{aligned}$$

$$\begin{aligned} D^2\Lambda _{\varvec{\rho }}^*(\varvec{\lambda })= D\log \varvec{\Phi }(\varvec{\lambda })=D^2S(\varvec{\lambda }),\quad \varvec{\lambda }\in A, \end{aligned}$$

where *S* is the thermodynamic entropy. Since $\Lambda _{\varvec{\rho }}^*$ and its derivative vanish at $\varvec{\rho }$, by Taylor expansion of $\Lambda _{\varvec{\rho }}^*$ around $\varvec{\rho }\in K$

$$\begin{aligned} \Lambda _{\varvec{\rho }}^*(\varvec{\lambda })=\int _0^1(1-t)\big \langle \varvec{\lambda } -\varvec{\rho },D^2S(\varvec{\gamma }_{\varvec{\rho },\varvec{\lambda }}(t))(\varvec{\lambda } -\varvec{\rho })\big \rangle dt,\quad \lambda \in A. \end{aligned}$$

Denoting by $\lambda _-(\varvec{\rho })>0$ the minimal eigenvalue of the strictly positive definite matrix $D^2S(\varvec{\rho })$, we have by continuity that

$$\begin{aligned} B:=\inf _{\varvec{\rho }\in K_\varepsilon }\lambda _-(\varvec{\rho })>0. \end{aligned}$$

Then $\Lambda _{\varvec{\rho }}^*(\varvec{\lambda })\ge \frac{B}{2}|\varvec{\lambda } -\varvec{\rho }|^2$ for all $(\varvec{\rho },\varvec{\lambda })\in \mathcal E_0^\varepsilon$, which shows that

$$\begin{aligned} \sup _{(\varvec{\rho },\varvec{\lambda })\in \mathcal E_0^\varepsilon }\frac{|\Psi _i(\varvec{\rho }, \varvec{\lambda })|}{\Lambda _{\varvec{\rho }}(\varvec{\lambda })}\le \frac{A^i}{B}<+\infty ,\quad i=1,2 \end{aligned}$$

and yields the bound (61) in the region $\mathcal {E}_0^\varepsilon$.

It remains to show that the supremum is finite in the region $\mathcal {E}_M^\infty$ for some $M>0$. On one hand, it follows from (16) and the compactness of *K* that $\varvec{\Psi }$ satisfies a bound of the form

$$\begin{aligned} |\varvec{\Psi }(\varvec{\rho },\varvec{\lambda })|_1\le C_0+C_1|\varvec{\lambda }|_1\quad \forall \;(\varvec{\rho },\varvec{\lambda })\in K\times \mathbbm {R}_+^2 \end{aligned}$$

for some constants $C_0,C_1\ge 0$. So, to complete the proof, it suffices to show that $\Lambda ^*$ has at least linear growth in $\mathcal E_M^\infty$ as $|\varvec{\lambda }|\rightarrow +\infty$, i.e.,

$$\begin{aligned} \lim _{M\rightarrow +\infty }\inf _{(\varvec{\rho },\varvec{\lambda })\in \mathcal E_M^\infty } \frac{\Lambda ^*_{\varvec{\rho }}(\varvec{\lambda })}{|\varvec{\lambda }|}>0, \end{aligned}$$

where of course the limit as $M\rightarrow +\infty$ exists as an increasing limit. We begin by noting that

$$\begin{aligned} \lim _{M\rightarrow +\infty }\inf _{(\varvec{\rho },\varvec{\lambda })\in \mathcal E_M^\infty } \frac{\Lambda ^*_{\varvec{\rho }}(\varvec{\lambda })}{|\varvec{\lambda }|} \ge \liminf _{|\lambda |\rightarrow +\infty }\inf _{\varvec{\rho }\in K}\frac{\Lambda _{\varvec{\rho }}^*(\varvec{\lambda })}{|\varvec{\lambda }|}=:a. \end{aligned}$$

We choose a sequence $\{\varvec{\lambda }_n\}\subseteq \mathbbm {R}_+^2$ achieving the limit inferior,

$$\begin{aligned} |\varvec{\lambda }_n|\rightarrow +\infty \quad \text {and}\quad \lim _{n\rightarrow +\infty } \inf _{\varvec{\rho }\in K} \frac{\Lambda _{\varvec{\rho }}^*(\varvec{\lambda }_n)}{|\varvec{\lambda }_n|}=a. \end{aligned}$$

Since $\{\frac{\varvec{\lambda }_n}{|\varvec{\lambda }_n|}\}$ is contained in the compact space $S^1_+:=S^1\cap \mathbbm {R}_+^2$, by passing to a subsequence if necessary, we can assume that $\{\frac{\varvec{\lambda }_n}{|\varvec{\lambda }_n|}\}$ converges to some direction $\varvec{\upsilon }\in S^1_+$. Then obviously

63 $$\begin{aligned} \liminf _{n\rightarrow +\infty }\frac{S(\varvec{\lambda }_n)}{|\varvec{\lambda }_n|}\ge \liminf _{\begin{array}{c} |\varvec{\lambda }|\rightarrow +\infty \\ {\varvec{\lambda }}/{|\varvec{\lambda }|} \rightarrow \upsilon \end{array}}\frac{S(\varvec{\lambda })}{|\varvec{\lambda }|} =S_\infty (\varvec{\upsilon }), \end{aligned}$$

where the equality in the right-hand side holds by (58). Since $\varvec{\Phi }(K)\subseteq \mathcal D_Z^o\cap (0,\infty )^2$, we have by (60) that $S_\infty (\varvec{\upsilon })-\langle \varvec{\upsilon },\log \varvec{\Phi }(\varvec{\rho })\rangle >0$ for all $\varvec{\rho }\in K$. Thus, since $\varvec{\Phi }$ is continuous and *K* is compact,

$$\begin{aligned} \theta :=\inf _{\varvec{\rho }\in K}\{S_\infty (\varvec{\upsilon })-\langle \varvec{\upsilon },\log \varvec{\Phi }(\varvec{\rho })\}>0. \end{aligned}$$

Then by (63) there exists $n_1\in \mathbbm {N}$ such that

$$\begin{aligned} n\ge n_1\quad \Longrightarrow \quad \frac{S(\varvec{\lambda }_n)}{|\varvec{\lambda }_n|}\ge S_\infty (\varvec{\upsilon })-\frac{\theta }{3}. \end{aligned}$$

By (54) and taking into account the fact that $Z\ge 1$, we have that for all $n\ge n_1$ and all $\varvec{\rho }\in K$,

$$\begin{aligned} \frac{\Lambda _{\varvec{\rho }}^*(\varvec{\lambda }_n)}{|\varvec{\lambda }_n|}&\ge S_\infty (\varvec{\upsilon })-\Big \langle \frac{\varvec{\lambda }_n}{|\varvec{\lambda }_n|}, \log \varvec{\Phi }(\varvec{\rho })\Big \rangle +\frac{1}{|\varvec{\lambda }_n|}\log Z\big (\varvec{\Phi }(\varvec{\rho })\big )-\frac{\theta }{3}\\&\ge S_\infty (\varvec{\upsilon })-\Big \langle \frac{\varvec{\lambda }_n}{|\varvec{\lambda }_n|}, \log \varvec{\Phi }(\varvec{\rho })\Big \rangle -\frac{\theta }{3}. \end{aligned}$$

But by the compactness of *K*, we have that $\Vert \log \varvec{\Phi }\Vert _{L^\infty (K)}:=\sup _{\varvec{\rho }\in K}|\log \varvec{\Phi }(\varvec{\rho })|_2<+\infty$ and therefore the sequence $\{\langle \frac{\varvec{\lambda }_n}{|\varvec{\lambda }_n|},\log \varvec{\Phi }(\varvec{\rho })\rangle \}$ converges to $\langle \varvec{\upsilon },\log \varvec{\Phi }(\varvec{\rho })\rangle$ uniformly over all $\varvec{\rho }\in K$,

$$\begin{aligned} \sup _{\varvec{\rho }\in K}\Big |\Big \langle \frac{\varvec{\lambda }_n}{|\varvec{\lambda }_n|}, \log \varvec{\Phi }(\varvec{\rho })\Big \rangle -\big \langle \varvec{\upsilon },\log \varvec{\Phi }(\varvec{\rho }) \big \rangle \Big | \le \Vert \log \varvec{\Phi }\Vert _{L^\infty (K)}\Big |\frac{\varvec{\lambda }_n}{|\varvec{\lambda }_n|}-\varvec{\upsilon }\Big |_2\rightarrow 0. \end{aligned}$$

Therefore we can choose $n_2\in \mathbbm {N}$ such that

$$\begin{aligned} n\ge n_2\quad \Longrightarrow \quad \sup _{\varvec{\rho }\in K}\Big |\Big \langle \frac{\varvec{\lambda }_n}{|\varvec{\lambda }_n|}, \log \varvec{\Phi }(\varvec{\rho })\Big \rangle -\big \langle \varvec{\upsilon },\log \varvec{\Phi }(\varvec{\rho })\big \rangle \Big |<\frac{\theta }{3}, \end{aligned}$$

and then for all $n\ge n_1\vee n_2$ and all $\varvec{\rho }\in K$

$$\begin{aligned} \frac{\Lambda _{\varvec{\rho }}^*(\varvec{\lambda }_n)}{|\varvec{\lambda }_n|}\ge S_\infty (\varvec{\upsilon })-\Big \langle \varvec{\upsilon },\log \varvec{\Phi }(\varvec{\rho }) \Big \rangle -\frac{2\theta }{3} \ge \theta -\frac{2\theta }{3}=\frac{\theta }{3}>0. \end{aligned}$$

This proves that

$$\begin{aligned} \liminf _{|\varvec{\lambda }|\rightarrow +\infty }\inf _{\varvec{\rho }\in K}\frac{\Lambda _{\varvec{\rho }}^*(\varvec{\lambda })}{|\varvec{\lambda }|} =\lim _{n\rightarrow +\infty }\inf _{\varvec{\rho }\in K}\frac{\Lambda _{\varvec{\rho }}^*(\varvec{\lambda }_n)}{|\varvec{\lambda }_n|}>0, \end{aligned}$$

which completes the proof of Lemma 4.5. $\square$

Since Lemma 4.5 establishes the missing bound (57), the proof of Theorem 3.2 is complete.

### Proof of Theorem 3.3

By [1], it is known that quasilinear parabolic systems have unique maximal classical solutions when starting from initial profiles of class $C^{2+\theta }$, $\theta \in [0,1)$. To show that classical solutions of the species-blind parabolic system (26) are global in time, we prove first a maximum principle asserting that the region

$$\begin{aligned} A:=\varvec{R}(\mathcal D_{\varvec{R}}^o)\cap (0,\infty )^2=\big \{\varvec{\rho }\in (0,\infty )^2 \bigm |\rho _1+\rho _2<\hat{\rho }_c\big \} \end{aligned}$$

is invariant under the evolution of the species-blind parabolic system. Here $\hat{\rho }_c\in (0,+\infty ]$ is the critical density of the one-species ZRP associated with the species-blind ZRP. The proof of this version of the maximum principle for systems of the form (26) relies on the maximum principle for quasilinear PDEs in divergence form found in [2]. Since, as we will see, the solution $\varvec{\rho }$ cannot lose regularity, we will obtain the existence of global in time classical solutions.

#### Lemma 4.6

(A maximum principle for the species-blind parabolic system) Let $\varvec{\rho } = (\rho _1,\rho _2)\in C^{1,2}([0,T)\times \mathbbm {T}^d;\mathbbm {R}^2)$, $T > 0$, be a classical solution of the species-blind parabolic system (26) starting from an initial condition $\varvec{\rho }_0\in C^2(\mathbbm {T}^d;\mathbbm {R}^2_+)$ satisfying

64 $$\begin{aligned} \varvec{\rho }_0(\mathbbm {T}^d)\subseteq A = \big \{\varvec{\rho }\in (0,\infty )^2\bigm |\rho _1 + \rho _2 < \hat{\rho }_c\big \}, \end{aligned}$$

where $\hat{\rho }_c$ is the critical density corresponding to the one-species density function $\hat{R}$. Then

65 $$\begin{aligned} 0&< \inf _{(t,u)\in [0;T)\times \mathbbm {T}^d}\rho _1(t,u)\wedge \rho _2(t,u)\nonumber \\&\le \sup _{(t,u)\in [0,T)\times \mathbbm {T}^d} \big (\rho _1(t,u)+\rho _2(t,u)\big )\le \sup _{u\in \mathbbm {T}^d} \big (\rho _1(0,u)+\rho _2(0,u)\big )<\hat{\rho }_c. \end{aligned}$$

#### Proof

By the continuity of $\varvec{\rho }_0$ and the compactness of $\mathbbm {T}^d$, there exists by assumption (64) an $\varepsilon >0$ such that

66 $$\begin{aligned} \varvec{\rho }_0(\mathbbm {T}^d)\subseteq \{(\rho _1,\rho _2)\in \mathbbm {R}^2 \bigm | \rho _1\wedge \rho _2 > \varepsilon ,\rho _1+\rho _2< \hat{\rho }_c-\varepsilon \}, \end{aligned}$$

where we replace $\hat{\rho }_c-\varepsilon$ by $\frac{1}{\varepsilon }$ when $\hat{\rho }_c=+\infty$. Since $\varvec{\rho }$ solves (26), by summing the two equations we see that the function $\rho _1+\rho _2$ solves the equation $\partial _t\rho = \Delta \hat{\Phi }(\rho )$. But since $\hat{\Phi }$ is the mean jump rate of a single species ZRP,

67 $$\begin{aligned} 0< c<\hat{\Phi }'(\rho )< C < +\infty \quad \text { for all } \rho \in [0,\hat{\rho }_c-\varepsilon /2] \end{aligned}$$

for some constants $c,C \ge 0$ and therefore the equation $\partial _t\rho = \Delta \hat{\Phi }(\rho )$ is uniformly parabolic, when considered for sub-critical initial conditions $\rho _0\in C(\mathbbm {T}^d,(0,\hat{\rho }_c))$. Therefore it follows by (66) and the maximum principle for scalar uniformly parabolic quasilinear equations that

68 $$\begin{aligned} 2\varepsilon<\inf _{(t,u)\in [0;T)\times \mathbbm {T}^d}(\rho _1+\rho _2)(t,u)\le \sup _{(t,u) \in [0;T)\times \mathbbm {T}^d}(\rho _1+\rho _2)(t,u)<\hat{\rho }_c-\varepsilon . \end{aligned}$$

We consider now the family of the open domains

$$\begin{aligned} B_\delta :=\{(\rho _1,\rho _2)\in \mathbbm {R}^2|\varepsilon<\rho _1+\rho _2<\hat{\rho }_c-\varepsilon , \;\rho _1\wedge \rho _2>-\delta \} \end{aligned}$$

for $\delta \in [0,+\infty ]$ and set

$$\begin{aligned} D_\delta :=\{(t,u,r)\in [0,T)\times \mathbbm {T}^d\times \mathbbm {R}|(r,\rho _2(t,u))\in B_\delta \}. \end{aligned}$$

Let $\Psi :D_\infty \rightarrow \mathbbm {R}$ denote the function given by the formula

$$\begin{aligned} \Psi (t,u,r)=r\frac{\hat{\Phi }(r+\rho _2(t,u))}{r+\rho _2(t,u)}. \end{aligned}$$

The sets $D_\delta$ are obviously open and the function $\Psi$ is well defined on $D_\infty$. Since the sum $\rho _1+\rho _2$ satisfies (68), we have that

$$\begin{aligned} (t,u,\rho _1(t,u))\in D_\infty \quad \text {for all }(t,u)\in [0,T)\times \mathbbm {T}^d, \end{aligned}$$

and since $(\rho _1,\rho _2)$ is a solution of (26), we have that $\rho _1$ solves

$$\begin{aligned} \partial _t\rho _1(t,u)=\Delta \Psi \big (t,u,\rho _1(t,u)\big ). \end{aligned}$$

In divergence form, the problem above is written as

69 $$\begin{aligned} \partial _t\rho _1(t,u)={\mathrm{div}}A_\Psi \big (t,u,\rho _1(t,u),\nabla \rho _1(t,u)\big ) \end{aligned}$$

where $A_\Psi :D_\infty \rightarrow \mathbbm {R}^d$ is the function given by the formula

$$\begin{aligned} A_\Psi (t,u,r,\upsilon )=\nabla _u\Psi (t,u,r)+\partial _r\Psi (t,u,r)\upsilon . \end{aligned}$$

Since $\rho _2$ is $C^{1,2}$, it follows that the function $A_\Psi$ is $C^1$ and $\partial _\upsilon A_\Psi (t,u,r,\upsilon )=\partial _r\Psi (t,u,r)I$ where $I\in \mathbbm {R}^{d\times d}$ denotes the identity matrix. By a simple calculation, $\partial _r\Psi (t,u,r)=H(r,\rho _2(t,u))$, where $H:B_\infty \rightarrow \mathbbm {R}$ is given by

$$\begin{aligned} H(\rho _1,\rho _2)=\frac{\rho _2}{\rho _1+\rho _2}\frac{\hat{\Phi }(\rho _1 +\rho _2)}{\rho _1+\rho _2} +\frac{\rho _1}{\rho _1+\rho _2}\hat{\Phi }'(\rho _1+\rho _2). \end{aligned}$$

We have that

70 $$\begin{aligned} \inf _{B_\delta }H\le \inf _{D_\delta }\partial _r\Psi \le \sup _{D_\delta } \partial _r\Psi \le \sup _{B_\delta }H \end{aligned}$$

for all $\delta \in [0,+\infty ]$ and it is obvious that

$$\begin{aligned} c\le \inf _{B_0}H\le \sup _{B_0}H\le C, \end{aligned}$$

where $c,C\ge 0$ are the constants in (67). By continuity of *H*, we obtain the existence of $\delta _0 > 0$ such that

71 $$\begin{aligned} \frac{c}{2}<\inf _{B_{\delta _0}}H\le \sup _{B_{\delta _0}}H<2C, \end{aligned}$$

which shows that the diagonal matrix $\partial _\upsilon A_\Psi$ is positive definite on the set $D_{\delta _0}\times \mathbbm {R}^d$. We set now

$$\begin{aligned} T^i:=\sup \Big \{t\in [0,T]\Big |\inf _{(s,u)\in [0,t)\times \mathbbm {T}^d} \rho _i(s,u)>0\Big \},\quad i=1,2. \end{aligned}$$

By the assumptions on the initial condition $\varvec{\rho }_0$, the set over which we take the supremum is non-empty. By the continuity of the solution $\varvec{\rho }$, we have $T^i > 0$ for $i = 1, 2$ and if $T^i < T$ then there exists $u_0^i\in \mathbbm {T}^d$ such that $\rho _i(T^i,u_0^i) = 0$. In order to prove the claim of the lemma, it suffices to show that $T^1 = T^2 = T$.

So we suppose that this is not true to obtain a contradiction. Without loss of generality it suffices to consider the cases $T^1< T^2 < T$ and $T^0 := T^1 = T^2 < T$.

- $T^1<T^2<T$: Since $\rho _1(t,u)\ge 0$ for all $(t,u)\in [0,T^1]\times \mathbbm {T}^d$ and $\rho _1$ is continuous in $[0,T)\times \mathbbm {T}^d$, there exists $t_0 > 0$ such that
  $$\begin{aligned} \inf _{(t,u)\in [0,T^1+t_0]\times \mathbbm {T}^d}\rho _1(t,u) > -\delta _0. \end{aligned}$$
  But then $(t,u,\rho _1(t; u))\in D_{\delta _0}$ for all $(t,u) \in [0,T^1 + t_0]\times \mathbbm {T}^d$ and so, since $\rho _1$ and 0 are solutions of problem (69) in $[0, T^1 + t_0] \times \mathbbm {T}^d$, which is uniformly parabolic in this region by (70) and (71), and since $\rho _1(T^1,u^1_0) = 0$, we get from [2, Theorem 1] that $\rho _1 \equiv 0$ in $[0,T^1) \times \mathbbm {T}^d$, which contradicts the definition of $T^1$.
- $T^0 := T^1 = T^2 < T$: Again, since $\rho _1(t,u)\wedge \rho _2(t,u)>0$ for all $(t,u)\in [0,T^0]\times \mathbbm {T}^d$, there exists $t_0 > 0$ such that
  $$\begin{aligned} \inf _{(t,u)\in [0,T^1+t_0]\times \mathbbm {T}^d}[\rho _1(t,u)\wedge \rho _2(t,u)]\ge -\delta _0. \end{aligned}$$
  But then again the problem (69) is uniformly parabolic in $[0,T^0 +t_0]\times \mathbbm {T}^d$ and $\rho _1$ and 0 are solutions with $\rho _1 \ge 0$ in $[0,T^0]$, which again by [2, Theorem 1] yields $\rho _1\equiv 0$ in $[0,T^0)\times \mathbbm {T}^d$ and contradicts the definition of $T^0$. $\square$

Using this maximum principle and the global existence of scalar uniformly parabolic equations, we obtain the global existence of solutions to the species-blind parabolic system as follows. To derive a contradiction, we assume that $\varvec{\rho }\in C^{1,2}([0,T_{\mathrm{max}})\times \mathbbm {T}^d;\mathbbm {R}^2)$, $T_{\mathrm{max}}<+\infty$, is the maximal classical solution of the species-blind parabolic system starting from $\varvec{\rho }_0\in C^{2+\theta }(\mathbbm {T}^d)$. Here maximality of the solution means that $\varvec{\rho }$ can not be extended to a $C^{1,2}$-solution on $[0,T)\times \mathbbm {T}^d$ for $T>T_{\mathrm{max}}$. Since $\hat{\rho }_0 := \rho _{01}+\rho _{02}\in C^{1+\theta ;2+\theta }(\mathbbm {T}^d;(0,\rho _c))$, there exists a unique solution $\hat{\rho }\in C^{1+\theta ,2+\theta }(\mathbbm {R}_+\times \mathbbm {T}^d;(0,\rho _c))$ of the scalar quasilinear parabolic equation $\partial _t\rho = \Delta \hat{\Phi }(\rho )$ with initial data $\hat{\rho }_0$. Then, $\hat{\rho }(\mathbbm {R}_+\times \mathbbm {T}^d)\subseteq (\varepsilon ,\hat{\rho }_c-\varepsilon )$ for some $\varepsilon > 0$ and the function $\phi (x) := \frac{\hat{\Phi }(x)}{x}$ is $C^\infty$ in $[\varepsilon , \hat{\rho }_c-\varepsilon ]$. Thus the function $a:\mathbbm {R}_+\times \mathbbm {T}^d\rightarrow \mathbbm {R}_+$ defined by $a(t,u):=\frac{\hat{\Phi }(\hat{\rho }(t,u))}{\hat{\rho }(t,u)}$ belongs to $C^{1+\theta ,2+\theta }(\mathbbm {R}_+\times \mathbbm {T}^d)$. Since $\hat{\Phi }'$ satisfies (67),

72 $$\begin{aligned} 0<c<a(t,u)\le C<+\infty \quad \text {for all }(t,u)\in \mathbbm {R}_+\times \mathbbm {T}^d \end{aligned}$$

for some constants $c,C\ge 0$. Since the function $\rho _1 + \rho _2$ is also a solution of the scalar equation $\partial _t\rho = \Delta \hat{\Phi }(\rho )$ with the same initial data $\rho _0$, we have by the uniqueness of solutions that

73 $$\begin{aligned} a\equiv \frac{\hat{\Phi }(\rho _1+\rho _2)}{\rho _1+\rho _2}\quad \text {in }[0,T_{\mathrm{max}})\times \mathbbm {T}^d. \end{aligned}$$

We consider the system

74 $$\begin{aligned} {\left\{ \begin{array}{ll} \partial _t\rho _1=\Delta \big (a(t,u)\rho _1(t,u)\big )\\ \partial _t\rho _2=\Delta \big (a(t,u)\rho _2(t,u)\big ) \end{array}\right. },\quad \varvec{\rho }(0,\cdot )=(\rho _{01},\rho _{02})\quad \text {in }\mathbbm {T}^d, \end{aligned}$$

which is obviously decoupled and can be solved by solving the scalar linear second order parabolic equation

75 $$\begin{aligned} \partial _t\rho =\Delta \big (a(t,u)\rho (t,u)\big ) \end{aligned}$$

twice with initial conditions $\rho _{01}$ and $\rho _{02}$. This scalar equation is given in general form by

$$\begin{aligned} \partial _t\rho =\sum _{i,j=1}^da^{ij}\partial _{ij}^2\rho +\sum _{i=1}^db^i\partial _i\rho +c\rho , \end{aligned}$$

where $a^{ij}=a\delta _{ij}$, $b^i=\partial _ia$ and $c=\Delta a$. We note that since *a* satisfies (72) and $a^{ij} = a\delta _{ij}$, the matrix $(a^{ij})$ is uniformly elliptic. Also, since $a\in C^{1+\theta ,2+\theta }(\mathbbm {R}_+\times \mathbbm {T}^d)$, the coefficients $a^{ij},b^i,c$ are $\theta$-Hölder continuous and so by the interpretation of [20, Theorem 5.14] in the flat torus with periodic boundary conditions, we find that for any $\rho _0 \in C^{2+\theta }(\mathbbm {T}^d)$ there exists a unique solution $\rho \in C^{1+\theta ,2+\theta }_{\mathrm{loc}}(\mathbbm {R}_+\times \mathbbm {T}^d)$ to the scalar problem (75) with initial condition $\rho _0$, and thus there exists a unique solution $\widetilde{\varvec{\rho }}\in C^{1+\theta ,2+\theta }_{\mathrm{loc}}(\mathbbm {R}_+\times \mathbbm {T}^d;\mathbbm {R}^2)$ of system (74) starting from $\varvec{\rho }_0 = (\rho _{01},\rho _{02})$. Since by (73), we have that the solution $\varvec{\rho }\in C^{1,2}([0,T_{\mathrm{max}})\times \mathbbm {T}^d;\mathbbm {R}^2)$ of the system (26) also solves the system (74), it follows by the uniqueness of solutions that $\widetilde{\varvec{\rho }}=\varvec{\rho }$ in $[0,T_{\mathrm{max}})\times \mathbbm {T}^d$. This, taking also into account the maximum principle, shows that

$$\begin{aligned} \varvec{\rho }\in C^{1+\theta ,2+\theta }([0,T_{\mathrm{max}})\times \mathbbm {T}^d;A). \end{aligned}$$

Now, we obviously have that $\widetilde{\varvec{\rho }}_{T_{\max }}\in C^{2+\theta }(\mathbbm {T}^d)$, and since $\widetilde{\varvec{\rho }}$ solves (26) in $[0,T_{\mathrm{max}})\times \mathbbm {T}^d$, we have by the maximum principle that

$$\begin{aligned} \widetilde{\varvec{\rho }}([0,T_{\mathrm{max}})\times \mathbbm {T}^d)\subseteq \{\varvec{r}\in A|d(\varvec{r},\partial A)>\delta \} \end{aligned}$$

for some $\delta >0$. Consequently, by continuity, we also have that $\widetilde{\varvec{\rho }}_{T_{\mathrm{max}}}(\mathbbm {T}^d)\subseteq A$. We consider then a solution $\varvec{r}:[0,\varepsilon )\times \mathbbm {T}^d\rightarrow A$, $\varepsilon >0$, of the problem (26) starting from $\varvec{r}_0=\widetilde{\varvec{\rho }}_{T_{\mathrm{max}}}$ and extend $\varvec{\rho }$ on $[0,T_{\mathrm{max}}+\varepsilon )\times \mathbbm {T}^d$ by defining $\varvec{\rho }(t,\cdot ):=\varvec{r}(t-T_{\mathrm{max}},\cdot )$ for $t\in [T_{\mathrm{max}},T_{\mathrm{max}}+\varepsilon )$. This function is obviously of class $C^{1+\theta ,2+\theta }$ and solves (26), which contradicts the maximality of $T_{\mathrm{max}}$. $\square$

### Proof of Corollary 3.1

By the global existence in time of solutions to the species-blind parabolic system, it suffices to check that Theorem 3.2 applies. Since the the one-species partition function $\hat{Z}$ is continuous on $\mathcal D_{\hat{Z}}$, it follows by the formula $Z(\varvec{\varphi })=\hat{Z}(\varphi _1+\varphi _2)$ that the partition function is continuous. It remains to check that in the case where the associated one-species ZRP has finite critical density, $\varvec{g}$ has regular tails, i.e., that for every $\varvec{\upsilon }\in S^1_{1,+}$

76 $$\begin{aligned} \mu _{c;1}(\varvec{\upsilon }):=\log \varphi _{c;1}(\varvec{\upsilon }) :=\liminf _{\begin{array}{c} |\varvec{k}|_1\rightarrow +\infty \\ \varvec{k}/|\varvec{k}|_1\rightarrow \varvec{\upsilon } \end{array}} \frac{1}{|\varvec{k}|_1}\log \varvec{g}!(\varvec{k}),\quad \varvec{\upsilon }\in S^1_{1,+} \end{aligned}$$

exists as a limit and is a continuous function of the direction $\varvec{\upsilon }\in S^1_{1,+}$. By the formula of $\varvec{g}!$ we have that

77 $$\begin{aligned} \frac{1}{|\varvec{k}|_1}\log \varvec{g}!(\varvec{k})=\frac{1}{|\varvec{k}|_1}\log \frac{k_1!k_2!}{|\varvec{k}|_1!}+\frac{1}{|\varvec{k}|_1}\log \hat{g}!(|\varvec{k}|_1). \end{aligned}$$

The second term in the right hand side of (77) converges as $|\varvec{k}|_1\rightarrow +\infty$ to the critical chemical potential $\hat{\mu }_c=\log \hat{\varphi }_c$ of the one-species jump rate $\hat{g}$. Since by Stirling’s approximation $\lim _{k\rightarrow +\infty }\frac{k!}{\sqrt{2\pi k}(k/e)^k}=1$, we can replace the liminf of the first term in the right hand side of (77) by

78 $$\begin{aligned} \liminf _{\begin{array}{c} |\varvec{k}|_1\rightarrow +\infty \\ \varvec{k}/|\varvec{k}|_1\rightarrow \varvec{\upsilon } \end{array}} \frac{1}{|\varvec{k}|_1}\log \sqrt{2\pi } \frac{\sqrt{k_1}k_1^{k_1}\sqrt{k_2}k_2^{k_2}}{\sqrt{|\varvec{k}|_1}| \varvec{k}|_1^{|\varvec{k}|_1}}. \end{aligned}$$

This limit inferior exists as a limit and defines a continuous function of $\varvec{\upsilon }$. Indeed, for all $\varvec{k}\in \mathbbm {N}^2$ we have that

$$\begin{aligned} \frac{1}{|\varvec{k}|_1}\log \frac{\sqrt{k_1}k_1^{k_1}\sqrt{k_2}k_2^{k_2}}{\sqrt{|\varvec{k}|_1}|\varvec{k}|_1^{|\varvec{k}|_1}}= \frac{1}{|\varvec{k}|_1}\log \frac{\sqrt{k_1k_2}}{\sqrt{|\varvec{k}|_1}}+\log \Big (\frac{k_1}{|\varvec{k}|_1}\Big )^{\frac{k_1}{|\varvec{k}|_1}} +\log \Big (\frac{k_2}{|\varvec{k}|_1}\Big )^{\frac{k_2}{|\varvec{k}|_1}}, \end{aligned}$$

and it is easy to check that $\lim _{|\varvec{k}|\rightarrow +\infty }\frac{1}{|\varvec{k}|_1}\log \frac{\sqrt{k_1k_2}}{\sqrt{|\varvec{k}|_1}}=0$, so that

$$\begin{aligned} \mu _{c;1}(\varvec{\upsilon })=\lim _{\begin{array}{c} |\varvec{k}|_1\rightarrow +\infty \\ \begin{array}{c} \varvec{k}/ |\varvec{k}|_1\rightarrow \varvec{\upsilon }\\ k_1,k_2>0 \end{array} \end{array}} \Big [\log \Big (\frac{k_1}{|\varvec{k}|_1}\Big )^{\frac{k_1}{|\varvec{k}|_1}} +\log \Big (\frac{k_2}{|\varvec{k}|_1}\Big )^{\frac{k_2}{|\varvec{k}|_1}}+\log g!(|\varvec{k}|_1)^{\frac{1}{|\varvec{k}|_1}}\Big ]=\langle \varvec{\upsilon }, \log \varvec{\upsilon }\rangle +\mu _c, \end{aligned}$$

with the convention $\upsilon _i\log \upsilon _i=0$ if $\upsilon _i=0$ since $x\log x\rightarrow 0$ as $x\rightarrow 0$. Finally, points $\varvec{k}\in \mathbbm {N}_0^2$ with $k_i=0$ for some $i=1,2$ contribute to the limit only if $\varvec{\upsilon }=\varvec{e}_i$ for some $i=1,2$. For such points $\varvec{k}\in \mathbbm {N}_0^2$, we have $k_1!k_2!=|\varvec{k}|_1!$, and so the first term in the right hand side of (77) vanishes, which agrees with the fact that $\langle \varvec{\upsilon },\log \varvec{\upsilon }\rangle =0$ if $\varvec{\upsilon }=\varvec{e}_i$, $i=1,2$. This is to be expected, since in the directions $\varvec{\upsilon }=\varvec{e}_i$ with $i=1,2$ in the phase space we have only one of the two species of particles, which when on their own perform the underlying one-species ZRP with critical chemical potential $\hat{\mu }_c=\log \hat{\varphi }_c$. This completes the proof that $\varvec{g}$ has regular tails. $\square$
